# ATGL From iWAT and BAT Is Crucial for Cardiac Remodeling and Metabolism After Myocardial Ischemia/Reperfusion

**DOI:** 10.1002/cph4.70106

**Published:** 2026-02-04

**Authors:** Heba Zabri, Alisa Ucar, Luzhou Wang, Simone Gorressen, Richard Kretschmer, Daniel Gorski, Tobias Lautwein, Mirela Balan, Stefan Lehr, Andre Heinen, Axel Gödecke, Jens W. Fischer, Katharina Bottermann

**Affiliations:** ^1^ Institute for Pharmacology, Medical Faculty and University Hospital Düsseldorf Heinrich Heine University Düsseldorf Düsseldorf Germany; ^2^ Genomics & Transcriptomics Laboratory (GTL) of the Heinrich‐Heine University Düsseldorf Düsseldorf Germany; ^3^ Core Unit Bioinformatics (CUBI) Medical Faculty and University Hospital Düsseldorf, Heinrich Heine University Düsseldorf Düsseldorf Germany; ^4^ Institute for Clinical Biochemistry and Pathobiochemistry, German Diabetes Center (DDZ) Leibniz Center for Diabetes Research at Heinrich Heine University Düsseldorf Düsseldorf Germany; ^5^ German Center for Diabetes Research (DZD e.V.) Munchen‐Neuherberg Germany; ^6^ Institute for Cardiovascular Physiology Medical Faculty and University Hospital Düsseldorf, Heinrich Heine University Düsseldorf Düsseldorf Germany; ^7^ CARID‐Cardiovascular Research Institute Düsseldorf Medical Faculty and University Hospital Düsseldorf, Heinrich Heine University Düsseldorf Düsseldorf Germany

**Keywords:** adiponectin, ATGL, brown adipose tissue, cardiac ischemia/reperfusion, cardiac substrate metabolism, spatial transcriptomics, subcutaneous and visceral white adipose tissue

## Abstract

Adipose tissue ATGL has emerged as an important player in cardiovascular disease. Myocardial infarction is accompanied by sympathetic stimulation and activation of white adipose tissue and peripheral lipolysis. We therefore investigate here the role of adipocyte ATGL in a murine model of cardiac ischemia and reperfusion (I/R) by using an inducible, adipocyte specific KO of ATGL (iatATGL‐KO). Notably this led to successfully inhibited lipolysis during cardiac ischemia, and KO mice exhibited aggravated cardiac dysfunction and enhanced scar formation after 28 days I/R. This phenotype went along with multiple structural and molecular alterations mainly in the subcutaneous white adipose tissue depot (iWAT) and brown adipose tissue (BAT). The iatATGL‐KO mainly reduced BAT activation as well as adiponectin‐secretion. In the heart spatial transcriptomic analysis suggested higher mechanical stress in the remote myocardium, which went along with higher oxygen consumption rates (OCR) and higher dependency on glucose as substrate after 24 h I/R. Taken together, iatATGL‐KO hearts after I/R seem to be affected in multiple ways, such as a reduction in cardioprotective factors from iWAT and BAT as well as an oxygen wasting effect in the remote zone of the heart, which contribute to the worse outcome. This indicates a time and depot‐specific role of adipocyte ATGL in cardiac ischemia and reperfusion injury.

## Graphical abstract

Cardiac I/R activates adipose tissue. iatATGL‐KO increases adipocyte size and reduces lipogenesis in WAT, induces whitening and inflammation in BAT, and reduces circulating NEFA and adiponectin levels. As a consequence, cardiac function is reduced, scar formation enhanced, and remote myocardium shows elevated mechanical stress and oxygen consumption.

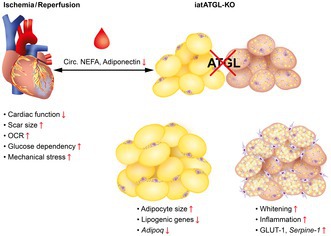

## 1 Introduction

Myocardial infarction (MI) is a disease with strong systemic implications. Due to neurohumoral activation of, for example, the sympathetic nervous system, the renin‐angiotensin‐aldosterone system, or natriuretic peptides (Remes 1994; Sigurdsson et al. 1993), peripheral organs such as the kidney, liver, or adipose tissue are affected by myocardial ischemia and the subsequent cardiac remodeling process. The remodeling process in the heart undergoes different inflammatory and reparative phases after cardiac ischemia, involving numerous cell types such as neutrophils and monocytes to clear the heart from necrotic cells and debris, macrophages to initiate healing, and fibroblasts and myofibroblasts to build the scar (Frangogiannis 2015).

It is long known that MI leads to elevated levels of circulating catecholamines and free fatty acids (FFA) (Gupta et al. 1969; Valori et al. 1967; Vetter et al. 1974). As catecholamines strongly activate lipolysis in white adipose tissue, these observations seem to be in causal relationship. However, this is a rather rapid and transient process, as FFA levels return to baseline levels within a few days (Petersen et al. 2003). We and others could show that the increase in FFA after cardiac ischemia is also present in murine models of myocardial infarction (Wang et al. 2022; Tian‐li et al. 2003). FFA are derived from triglyceride breakdown in adipocytes. The classical triglyceride breakdown cascade is stimulated via G_αs_‐protein coupled receptors, such as beta‐adrenergic receptors, after binding of ligands, such as catecholamines. The α‐subunit activates adenylate cyclase to produce cAMP, which activates protein kinase A (PKA). PKA has numerous targets, from which several are important for the lipolytic process, i.e., perilipin 1 (Plin1) and hormone sensitive lipase (HSL) (Young and Zechner 2013). The triglyceride breakdown is catalyzed by the three enzymes adipose triglyceride lipase (ATGL), HSL, and monoglyceride lipase (MGL).

Interestingly, we could demonstrate that cardiac ischemia/reperfusion (I/R) does not only activate lipolysis acutely during cardiac ischemia, but that white adipose tissue is affected chronically for up to 28 d after cardiac ischemia in a murine model. The alterations were depot‐specific and included browning of white adipocytes, inflammation, an altered expression profile of adipokines as well as reduced adipocyte size and an upregulation of the main lipolytic enzyme ATGL in the subcutaneous depot (Wang et al. 2022). ATGL was discovered roughly 20 years ago as the enzyme catalyzing the initial and rate‐limiting step of the triglyceride breakdown cascade, the hydrolysis of triacylglycerides (TAGs) to diacylglycerides (DAGs) and free fatty acids (Zimmermann et al. 2004; Jenkins et al. 2004; Villena et al. 2004; Haemmerle et al. 2006). ATGL is mainly expressed in white and brown adipose tissue and to a lesser extent in other organs such as liver, heart and skeletal muscle (Schreiber et al. 2019).

ATGL rapidly became an attractive therapeutic target, as specific inhibitors were developed (Mayer et al. 2013) and adipocyte‐specific KO‐mice showed improved glucose metabolism and protection from diet‐induced obesity (Raje et al. 2020; Schoiswohl et al. 2015). Also in the context of cardiovascular disease, the inhibition of adipocyte‐specific ATGL in mouse models via pharmacological or genetic tools was promising, as this resulted in improved cardiac function in response to pressure overload (Parajuli et al. 2018; Salatzki et al. 2018) or sympathetic cardiac stress (Takahara et al. 2021; Thiele et al. 2021). In an own study, we could also show that the application of the ATGL‐inhibitor Atglistatin before and during cardiac ischemia improved remote myocardial function 7 days post I/R in a murine model (Bottermann et al. 2020). However, in light of our findings that the subcutaneous WAT depot is also affected chronically during the cardiac remodeling process and upregulation of ATGL in the late reperfusion, we asked the question, which role adipocyte ATGL might play for adipose tissue (AT) and the heart after cardiac I/R. We therefore crossbred tamoxifen‐inducible, adipocyte‐specific ATGL‐KO mice and analyzed the different AT‐depots as well as cardiac function and metabolism at different time points after cardiac I/R. Surprisingly, we demonstrate a reduction in cardiac systolic function and a disturbed cardiac substrate metabolism, as well as severely disordered WAT and BAT function, indicating a distinct and specific role for adipocyte ATGL during the remodeling process after cardiac I/R.

## 2 Material and Methods

### 2.1 Mice

C57BL/6 J mice from Janvier Labs (France) and genetically modified iatATGL‐KO mice and their controls were used for the experiments. The genetically modified adipocyte‐specific and tamoxifen‐inducible KO of ATGL was obtained by crossing the ATGL‐flox line, generated by (Sitnick et al. 2013) (IMSR_JAX:024278), with the Adipoq‐CreERT2 line, generated by (Sassmann et al. 2010) (IMSR_JAX:025124). Both lines were obtained from The Jackson Laboratory (USA). Mice with Adipoq‐CreERT2^hemi^/ATGL^flox/flox^ were used as iat‐ATGL‐KO and with Adipoq‐CreERT2^wt/wt^/ATGL^flox/flox^ genotype as controls. The KO was induced by injecting 500 μg 4‐hydroxytamoxifen (Sigma‐Aldrich), suspended in peanut oil, intraperitoneally into animals aged approximately 10 weeks for 7 consecutive days, followed by a 2‐week washout period. Mice were housed at the central animal facility of the University Hospital Düsseldorf on a 12‐h day/night cycle with *ad libitum* access to water and food (V1184‐300, ssniff Spezialdiäten GmbH).

All animal experiments were performed according to national guidelines and approved by the authorities (LANUV, NRW, AZ 81‐02.04.2019.A397, AZ 2025‐172‐Grundantrag).

### 2.2 Myocardial Ischemia and Reperfusion

Male mice, aged 12–15 weeks, underwent myocardial ischemia for 60 min, followed by reperfusion for different durations (30 min, 24 h, 7 days, 28 days). Briefly, we used a closed chest model in a two‐step procedure (Nossuli et al. 2000). For ligature induction, mice were anesthetized with ketamine/xylazine (90–100 mg/kg/10–15 mg/kg BW), intubated and mechanically ventilated. Thorax was opened between the 3rd and 4th rib and the left anterior descending artery (LAD) was loosely ligated with a suture. A polyethylene tube (PE10) was threaded through both ends of the suture, left loose on the heart and the suture ends were kept in a subcutaneous pocket. Mice were allowed to recover 3–7 days. Myocardial ischemia was induced under 2% isoflurane by opening the skin and attaching 5 g weights to both ends of the suture, which were then suspended under tension for 60 min. Ischemia induction was verified by monitoring the ST‐segment elevation on the ECG using Basic Data Acquisition Software (Harvard Apparatus). Sham‐operated mice underwent the same procedure, but with no weights attached. Buprenorphine (0.05 mg/kg BW) was administered for intra‐ and postoperative analgesia.

### 2.3 Echocardiography

Cardiac function was determined via echocardiographic examinations using ultrasound device Vevo 3100 (Visual Sonics) and ultrasound probe MX400. Animals were anesthetized with 2%–3% isoflurane and fixed to a heated electrode contact pad to enable monitoring of heart and breathing rate, ECG and body temperature. Chest hair was removed before applying the gel. Measurements were taken of the left ventricle in the parasternal long axis in B‐ and M‐mode, and in the parasternal short axis in B‐mode at the middle, base, and apex. Cardiac volumes and functional parameters were determined using the Simpson's method. Strain analysis was performed using Vevo Strain Analysis software in VevoLAB 5.11.1.

### 2.4 Measurement of Non‐Esterified Fatty Acids

Blood samples were taken either from the tail vein or from the heart *post mortem* and serum was collected by centrifugation. The colorimetric NEFA‐HR(2) assay (FUJIFILM Wako) was used according to the manufacturer's protocol as described earlier (Wang et al. 2022).

### 2.5 CL‐316,243 Administration

In vivo ATGL activity was evaluated by stimulating lipolysis via intraperitoneal injection of the β_3_‐adrenoreceptor agonist CL‐316,243 (Sigma‐Aldrich) (1 mg/kg bodyweight) and measuring NEFA levels in serum from blood samples collected before and 30 min after administration.

### 2.6 Adipose Tissue Explants

Ex vivo ATGL activity was assessed by measuring the release of NEFAs from adipose tissue explants according to a modified protocol from (Schweiger et al. 2014). Inguinal and gonadal white adipose tissue, as well as brown adipose tissue, were excised, rinsed briefly with cold PBS, and transferred as fast as possible into 37°C pre‐warmed Gibco DMEM, low glucose (Thermo Fisher Scientific). Adipose tissue was cut into small pieces (~20 mg), and each piece was placed in 200 μL DMEM (37°C) in a 96‐well plate. Samples were then incubated in 200 μL DMEM supplemented with 2% fatty acid‐free BSA (Roche), either in presence or absence of 10 μM isoproterenol (Sigma‐Aldrich), at 37°C. After 1 h of incubation, medium was exchanged and adipose tissue was incubated for an additional hour under the same conditions. Following the second incubation, the tissue was removed and NEFA concentrations in the medium were measured.

### 2.7 Multiplex Analysis of Adipokines

Cardiac blood was used to determine the serum concentrations of leptin and adiponectin. Bio‐Plex Pro Mouse Diabetes 8‐Plex Assay and Bio‐Plex Pro Mouse Diabetes Adiponectin Assay (Bio‐Rad Laboratories Inc.) were performed according to the manufacturers' instructions and the results were measured using Bio‐Plex 200 System (Bio‐Rad Laboratories Inc.).

### 2.8 Histological Analysis

#### 2.8.1 Immunofluorescence

Immunofluorescence staining of paraffin embedded adipose tissue slices was performed as described before (Wang et al. 2022). In brief, sections were stained after deparaffinization, antigen retrieval, and blocking with Anti‐Mouse/Human Mac‐2 (Cedarlane), and anti‐UCP1 (Abcam) and Alexa Fluor 647‐labeled corresponding 2nd antibody (Thermo Fisher Scientific). Wheat germ agglutinin (WGA)‐Alexa Fluor 488 conjugate (Thermo Fisher Scientific) was added in 2nd antibody‐staining solution. Imaging was performed using Keyance BZ800 fluorescence microscope.

Immunofluorescence staining of 8μm thick cardiac cryosections was performed as described before (Emde et al. 2014) using WGA Alexa Fluor 488 conjugate (Thermo Fisher Scientific). Imaging was performed using Zeiss Imager.M2.

#### 2.8.2 H&E Staining and Determination of Adipocyte Size

Heat‐fixed, 5 μm thick, paraffin‐embedded sections of adipose tissue were deparaffinized in Roticlear (Carl Roth) for 3 × 15 min, then hydrated in a descending ethanol series (absolute, 96% and 70%) for 2 min each, followed by washing in PBS for 2 × 5 min, then in distilled water for 1 min. Sections were then incubated in hemalaun solution (Carl Roth) for 1 min, immersed in water, immersed in 1% HCl, blued for 10 min under running tap water and incubated in 1% eosin G solution (Carl Roth) for 1 min. Sections were then dehydrated using an ascending ethanol series for 2 min each and Roticlear for 5 min. Mounting was performed using Rotimount (Carl Roth). After microscoping with a 20× objective, sizes of 200 adipocytes were manually determined using Fiji (ImageJ) (Schindelin et al. 2012) by tracing their outlines.

#### 2.8.3 Evans Blue and 2,3,5‐Triphenyltetrazolium Chloride (TTC) Staining

24 h after reperfusion the myocardial infarct size was measured using Evans Blue/TCC dual staining. Mice were sacrificed, hearts excised and washed in ice‐cold saline/heparin solution. To distinguish the area at risk (AAR) from the remote area, LAD ligature was reclosed by replacing the old suture and injecting 1% Evans Blue solution (Sigma‐Aldrich) through the aortic root into the coronary arteries, resulting in a blue stained remote area. Then a second staining was performed to differentiate between viable and necrotic regions within the AAR. Hearts were frozen at −20°C for 30 min and cut into 1 mm thick slices, which were incubated for 3 min at 37°C in 1% TTC (Sigma‐Aldrich). Subsequently, sections were imaged, weighed and the different areas were determined using the Diskus Software.

### 2.9 RNA Isolation, Reverse Transcription and qPCR Analysis

RNA was isolated using RNeasy Lipid Tissue Mini Kit (adipose tissue) or RNeasy Fibrous Tissue Mini Kit (cardiac tissue) (Qiagen) according to the manufacturers' instructions. Qualitative and quantitative analysis was performed using spectrophotometer NanoDrop One (Thermo Fisher Scientific). RNA was transcribed into cDNA using a QuantiTect Reverse Transcription Kit (Qiagen) and a Mastercycler (Eppendorf). Quantitative mRNA gene expression was measured by qPCR using Platinum SYBR Green qPCR SuperMix‐UDG (Thermo Fisher Scientific) and primer sequences listed in Table 1 with Harvard Primer Bank ID (Wang and Seed 2003; Spandidos et al. 2008, 2010) when applicable. Primers were obtained either predesigned from Merck (KiCqStart‐Primer) or from Thermo Fisher Scientific. Amplification was performed using a StepOnePlus Real‐Time PCR System (Thermo Fisher Scientific). *Nudc* was used for normalization as a housekeeping gene. Relative gene expression levels were quantified using the ΔCt‐method by subtracting the Ct values of the housekeeping gene from the Ct values of the target gene and then calculating the resulting values as a negative power of 2. Finally, all values were divided by the mean of the control group.

**TABLE 1:** Sequences of used primers.

Gene	Sequence forward primer 5′→3′	Sequence reverse primer 5′→3′	Harvard Primer Bank ID
*Adipoq*	CCA CTT TCT CCT CAT TTC TG	CTA GCT CTT CAG TTG TAG TAA C	
*Cidea*	GTG TTA AGG AAT CTG CTG AG	CTA TAA CAG AGA GCA GGG TC	
*Cox8b*	ATC TCA GCC ATA GTC GTT G	CTG CGG AGC TCT TTT TAT AG	
*Dgat2*	GCG CTA CTT CCG AGA CTA CTT	GGG CCT TAT GCC AGG AAA CT	16975490a1
*Lep*	GAG ACC CCT GTG TCG GTT C	CTG CGT GTG TGA AAT GTC ATT G	6678678a1
*Lgals3*	GGA GAG GGA ATG ATG TTG CCT	TCC TGC TTC GTG TTA CAC ACA	225543162c1
*Nudc*	AGA ACT CCA AGC TAT CAG AC	CTT CAG GAT TTC CTG TTT CTT C	
*Pnpla2*	CAA CCT TCG CAA TCT CTA C	TTC AGT AGG CCA TTC CTC	
*Pparg*	AAA GAC AAC GGA CAA ATC AC	GGG ATA TTT TTG GCA TAC TCT G	
*Prdm16*	ATC TAC AGG GTA GAA AAG CG	TCT CCG TCA TGG TTT CTA TG	
*Serpine1*	TCT GGG AAA GGG TTC ACT TTA CC	GAC ACG CCA TAG GGA GAG AAG	170172561c1
*Slc2a1*	GCA TCT TCG AGA AGG CAG GT	GTC CAG CTC GCT CTA CAA CA	
*Srebf1*	TGA CCC GGC TAT TCC GTG A	CTG GGC TGA GCA ATA CAG TTC	13097057a1

### 2.10 Western Blot Analysis

Adipose tissue was lysed in 500 μL of lysis buffer (20 mM Tris–HCl, 1 mM EDTA and 255 mM sucrose, pH 7.4) containing Halt Protease and Phosphatase Inhibitor Cocktail (Thermo Fisher Scientific). Tissue was lysed using a TissueRuptor II (Qiagen), then centrifuged, and protein was collected from the middle phase supernatant. Protein concentration was quantified using Pierce BCA Protein Assay Kit (Thermo Fisher Scientific). To achieve the same concentrations, samples were diluted with 4× Laemmli buffer (250 mM Tris (pH = 6.8), 8% SDS, 20% glycerol, 0.02% bromophenol blue, 100 mM DTT) containing freshly prepared DTT. Proteins were denatured by incubation at 95°C for 5 min. For SDS‐polyacrylamide gel electrophoresis, 30–50 μg of protein and PageRuler Prestained Protein Ladder (Thermo Fischer Scientific) were loaded onto a 10% polyacrylamide gel. After separation, proteins were blotted from the gel to a nitrocellulose membrane using a semi‐dry blotter (Peqlab) at 110 mA for 60 min or Trans‐Blot Turbo Transfer System (Biorad) (10 min, 130 mA, 25 V). Total proteins were stained using Revert 700 Total Protein Stain and Wash Solution Kit (LI‐COR) and imaged. Non‐specific binding sites were blocked with a blocking buffer (LI‐COR Intercept Blocking Buffer (LI‐COR) in TBS 1:1) for 1 h. The membrane was then incubated overnight at 4°C with the primary antibodies: ATGL (30A4) Rabbit mAb, HSL (D6W5S) XP Rabbit mAb, Phospho‐HSL (Ser563) Antibody, Phospho‐PKA Substrate (RRXS*/T*) (100G7E) Rabbit mAb, or Glut1 (D3J3A) Rabbit mAb (all Cell Signaling Technologies). After washing with TBST (3 × 10 min), membrane was incubated with the secondary antibody (IRDye 800CW Goat anti‐Rabbit (LI‐COR)) for 1 h at room temperature. After washing with TBST (3 × 5 min), membrane was imaged using Odyssey Imaging System 9120 (LI‐COR). All signals were normalized to total protein stain according to the manufacturer's instructions, except for p‐HSL, which was normalized to total HSL.

### 2.11 Spatial Transcriptomics

The analysis was performed with *n* = 3 biological replicates.

Sample preparation: Hearts of iatATGL‐KO and control animals were harvested after 60 min ischemia and 24 h reperfusion, retrograde perfused with heparin/PBS solution, embedded in OCT and snap frozen in −30°C to –40°C cold isopentane. After RNA quality control from each tissue specimen, 10 μm sections were cut with a Leica CM3050 S cryotome (−20°C chamber temperature, −10°C object head temperature) and placed on 10× Genomics Visium gene expression slides for fresh frozen tissue. Methanol fixation and H&E staining were carried out according to the manufacturer's guidelines (Visium, 10× Genomics, Pleasanton, USA). Brightfield images were taken on a Zeiss Axio Imager.M2 microscope or a Keycance BZ‐800 with an 10× objective.

Library Generation: Prepared Visium slides were used as input for the Visium Spatial Gene Expression workflow (10× Genomics, Pleasanton, USA) according to the manufacturer's instructions. Tissue permeabilization was carried out for 12 min. Sequencing was performed on a NextSeq 2000 system (Illumina Inc., San Diego, USA) with a mean sequencing depth of ~40,000 reads/spot.

Spatial transcriptomics data processing: Raw sequencing data were processed using the 10× Genomics spaceranger software (v1.3.1). Aligning reads to the mm10 genome, UMI counting, tissue detection, and fiducial detection were performed via the spaceranger count pipeline.

The raw counts from the cellranger pipeline were processed using the Seurat R package (version 5.0.0) (Hao et al. 2024) as follows: After filtering out the low‐quality spots (nFeature < 300, nCount < 500) and genes expressed in less than 10 spots, the counts for each sample were normalized using the SCTransform function. Samples were then integrated using the CCA method. We performed dimensionality reduction followed by graph‐based clustering with a resolution of 0.4 using the functions RunPCA and FindClusters, respectively. The niches corresponding to tissue regions after a myocardial infarction (e.g., border zone, scar zone, etc.) were manually annotated, guided by each niche's top expressed marker genes (function: FindAllMarkers) and previously reported marker combinations (Calcagno et al. 2022; Kuppe et al. 2022; Yamada et al. 2022).

DEGs were detected using the FindMarkers function (log2FC > 0.25, p_val_adj < 0.05), sorted by log2 fold change of the average expression and visualized in Volcano plots for each cluster. All DEGs were used for over‐representation analysis with enrichGO (clusterProfiler v4.10.0, pvalueCutoff < 0.05) (Yu et al. 2012) and visualized as dotplot (enrichplot, v1.22.0) (Yu and Gao 2025).

Next, we calculated the average expression of each cluster for various gene sets using Seurat's AddModuleScore function to evaluate various signatures of interest using indicated gene ontology terms (Release 2021‐05‐01) (Ashburner et al. 2000; Carbon et al. 2009; The Gene Ontology Consortium et al. 2023). For visualization of gene expression comparatively between clusters and genotypes, as well as the spatial localization of gene expression, we used VlnPlot, DoHeatmap, SpatialFeaturePlot, and SpatialDimPlot of the Seurat package.

Finally, we used the Wilcoxon rank sum test with Bonferroni correction to account for the multiple testing problem to check for statistically significant differences in gene expression between clusters (rstatix v 0.7.2) (Kassambara 2023).

Differentially expressed genes of the remote niche were analyzed using QIAGEN Ingenuity Pathway Analysis (Krämer et al. 2014).

### 2.12 Extracellular Flux Measurement

Substrate metabolism was examined using extracellular flux measurements in the non‐ischemic remote area, represented by the intraventricular septum according to a modified protocol (Bottermann et al. 2023). After harvest, hearts were washed in PBS and the intraventricular septum was cut out and embedded in 4% warm (37°C) low melt agarose (Roth). After inserting the tissue into the agarose, sample was cooled down and the horizontally embedded septum was cut into 150 μm thick longitudinal sections in Tyrode buffer (18 mM NaHCO_3_, 126 mM NaCl, 4.4 mM KCl, 1 mM MgCl_2_, 4 mM Hepes, 11 mM glucose, 30 mM taurine and 10 mM 2,3‐butanedione monoxime) using a vibratome (1 mm amplitude, 85 Hz, 0.2–0.5 mm/s, VT1200S, Leica). Tissue sections were placed in ascending calcium concentrations (50 μM, 75 μM, 125 μM, 275 μM and 525 μM in Tyrode buffer) for 2 min each. Sections were then transferred to medium A (2 mM glutamine (Agilent), 5.5 mM glucose (Agilent), 0.5 mM carnitine (Sigma‐Aldrich) in DMEM) and tissue pieces were punched out using a sharpened cannula, placed in the wells of an Islet Capture Plate (Agilent) in 100 μL of medium B (2 mM glutamine, 5.5 mM glucose, 0.5 mM carnitine and 0.2 mM palmitate‐BSA in DMEM) and fixed with an islet capture screen. 400 μL of medium B (37°C) were added and the plate was incubated for 1 h at 37°C to allow equilibration. Subsequently, oxygen consumption was measured using a Seahorse XFe24 device (Agilent). Measurements were taken using a protocol consisting of cycles of 3 min of mixing, 2 min of waiting and 3 min of measuring. Oxygen consumption was measured at baseline and following the addition of mitochondrial uncoupler FCCP (3 μM, Sigma‐Aldrich), CPT1 inhibitor etomoxir (40 μM, Sigma‐Aldrich) and MPC inhibitor UK5099 (4 μM, Sigma‐Aldrich). Non‐mitochondrial oxygen consumption was determined by adding a combination of electron transport chain inhibitors: rotenone (3 μM, Sigma‐Aldrich) and antimycin A (10 μM, Sigma‐Aldrich). Mitochondrial oxygen consumption was determined by subtracting the non‐mitochondrial oxygen consumption values from the oxygen consumption values before inhibiting the mitochondrial electron transport chain.

### 2.13 Statistical Analysis

Statistical analyses were performed using GraphPad Prism 10 and the results are presented as mean ± standard error of the mean (SEM). Differences between two groups were tested using unpaired *t*‐test; Welch's correction was applied when necessary. Not normally distributed data were analyzed using the non‐parametric Mann–Whitney test. Differences between more than two groups were analyzed via one‐way ANOVA with Tukey's multiple comparisons test. Two‐way ANOVA followed by Sidak's multiple comparisons test was performed when two variables (genotype and time or cell size) were compared. A *p*‐value of less than 0.05 was considered statistically significant. Statistical tests used are detailed in the figure legends.

## 3 Results

### 3.1 iatATGL‐KO Mice Show Aggravated Cardiac Dysfunction and Larger Scars After Cardiac I/R

**FIGURE 1:**
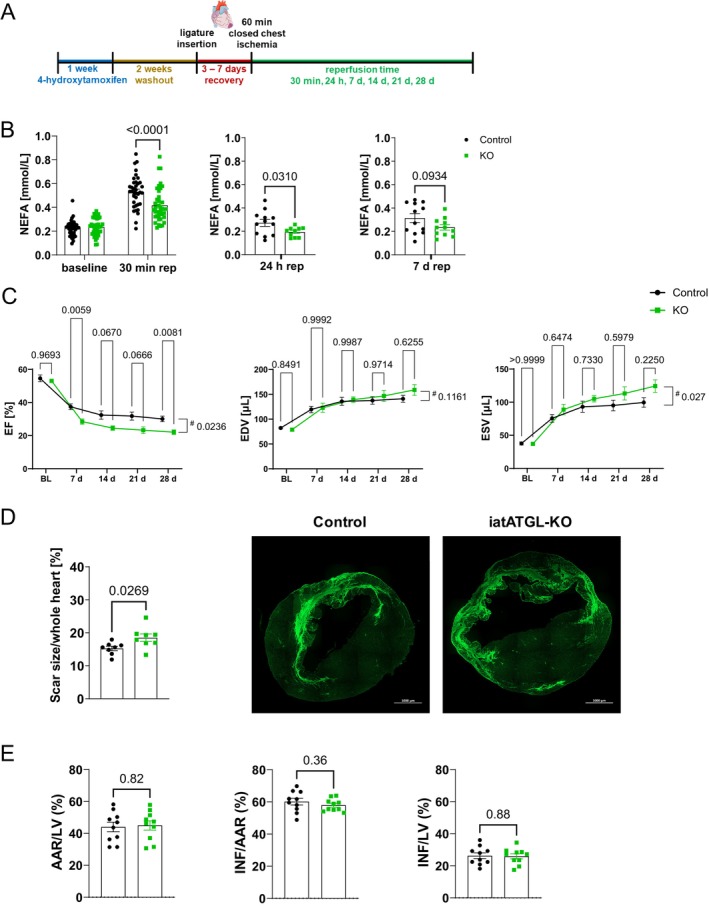
Cardiac phenotype of iatATGL‐KO mice after cardiac ischemia/reperfusion. (A) Timeline of treatment protocol. (B) Level of non‐esterified fatty acids (NEFA) in iatATGL‐KO (KO) and control mice before ischemia and after 60 min ischemia and 30 min reperfusion (repetitive sampling), after 24 h and after 7 days reperfusion. *n* = 37–40, two‐way ANOVA Sidaks multiple comparisons test for BL/30 min rep, *n* = 10–12, Welch's test for 24 h, *n* = 11–12 unpaired two‐tailed *t*‐test for 7 days. (C) Cardiac ejection fraction (EF), end diastolic and end systolic volume (EDV, ESV) before cardiac ischemia and weekly after 60 min ischemia in iatATGL‐KO and control mice. *n* = 11–12, two‐way ANOVA (#: *P* = time × genotype), Sidaks multiple comparisons test. (D) Cardiac scar size per whole heart area in KO and control mice after 28 days rep. Exemplary images of WGA staining. *n* = 8, unpaired two‐tailed *t*‐test. (E) Cardiac infarct size in KO and control mice after 24 h rep. Given is area at risk (AAR) per left ventricle (LV), infarct (INF) per AAR and INF per LV. *n* = 10, unpaired two‐tailed *t*‐test. All data are presented as mean ± SEM.

After KO‐induction and verification (Figure S1), mice underwent 60 min closed‐chest cardiac ischemia followed by 28 days reperfusion (rep) (Figure 1A). ATGL‐KO mice are known for lower levels of lipolytic products such as non‐esterified fatty acids (NEFA) (Schoiswohl et al. 2015). In previous work, we demonstrated elevated NEFA levels 30 min after cardiac ischemia in the here used mouse model of closed chest I/R (Wang et al. 2022). Additionally, we show here an increased phosphorylation of PKA substrates and HSL at Ser563 in the inguinal WAT depot (Figure S4), indicating iWAT as the major source of NEFAs after I/R. In iatATGL‐KO and control mice, blood was sampled before ischemia and after 30 min rep and additionally during harvest after 24 h and 7 days rep. Circulating NEFAs were measured and exhibited significantly lower levels compared to control after 30 min rep (ctrl 0.53 ± 0.02 mmol/L, KO 0.42 ± 0.02 mmol/L), 24 h rep and a trend towards lower levels after 7 days rep (Figure 1B), indicating a sufficient reduction in adipocyte ATGL activity, mainly when lipolysis is stimulated.

Cardiac function was measured before and weekly after I/R and showed an aggravation of cardiac systolic dysfunction following cardiac ischemia, as seen by a significant worsened ejection fraction (28 days: ctrl. 30.1% ± 1.7%, KO 22.1% ± 1.4%, *p* (time × genotype) = 0.02) and increased end systolic volume (28 days: ctrl. 99.5 ± 7.2 μL, KO 124.3 ± 9.4 μL, *p* (time × genotype) = 0.03) while end diastolic volume was unchanged (Figure 1C). This went along with a reduced global longitudinal strain (28 days: ctrl. −7.1% ± 0.6%, KO −5.1% ± 0.4%), while the assessment of regional radial and longitudinal peak strain revealed a significant reduction only in the anterior mid segment (longitudinal peak strain) of the left ventricle (Figure S3). To assess if the worsened cardiac function was due to a larger scar in the left ventricle, scar size was analyzed via WGA‐staining after 28 days I/R. KO animals had a significantly larger scar per whole heart area compared to control (Ctrl. 15.3% ± 0.7%, KO 18.5% ± 1.1%) (Figure 1D). Next, infarct size (IS) per area at risk (AAR) as well as per left ventricle was measured after 24 h I/R to quantify the contribution of the ischemic injury to the larger scar. AAR and IS were unchanged between the two groups (Figure 1E), thus, we concluded that iatATGL‐KO mice rather had stronger scar formation after I/R than a bigger ischemic injury.

### 3.2 iatATGL‐KO Mainly Alters iWAT and BAT

**FIGURE 2:**
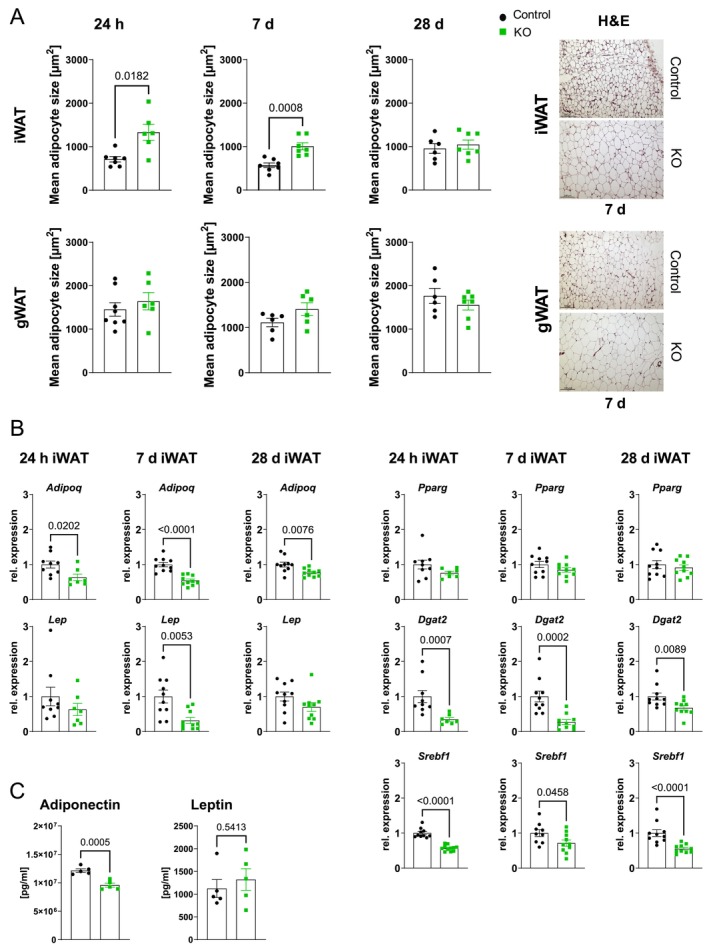
Phenotype of white adipose tissue in iatATGL‐KO mice after cardiac ischemia/reperfusion. (A) Mean adipocyte size in inguinal (iWAT) and gonadal (gWAT) white adipose tissue after 24 h, 7 days and 28 days I/R in iatATGL‐KO (KO) and control mice. Exemplary images of H&E stained iWAT and gWAT in KO and control (7 days rep). *n* = 6–8, unpaired two‐tailed *t*‐test and Welch's test. (B) Relative gene expression of adiponectin (*Adipoq*), leptin (*Lep*), *Pparγ*, *Dgat2* and Srebp1c (*Srebf1*) in iWAT of KO relative to control after 60′ ischemia and either 24 h, 7 days or 28 days rep. *n* = 7–10, unpaired two‐tailed *t*‐test, Welch's test, Mann–Whitney test. (C) Circulating level of adiponectin and leptin in KO and control after 7 d rep. *n* = 5, unpaired two‐tailed *t*‐test. All data are presented as mean ± SEM.

As the KO of ATGL was induced in adipose tissue we analyzed white and brown adipose tissue for alterations which might explain the observed cardiac phenotype. When measuring body weight and fat depot weights, it was striking to see that while body weight was not significantly changed between control and KO after I/R, especially iWAT/BW and BAT/BW ratio was increased. gWAT/BW ratio was significantly higher at 24 h and 7 days rep but not at 28 days rep (Figure S5A). We therefore analyzed again adipose tissue ATGL‐protein content at 28 days rep, which was still significantly lower in KO mice (Figure S6). In line with the tissue weights, also mean adipocyte size was significantly higher in iWAT of KO mice at 24 h and 7 d reperfusion, while there was no difference at 28 days reperfusion. In gWAT mean adipocyte size was unchanged at all reperfusion time points (Figure 2A, Figure S7). These differences between iWAT and gWAT were also reflected in gene expression analysis regarding adipokines and lipogenesis. Adiponectin expression was significantly downregulated in iWAT at all reperfusion time points, which was also mirrored by reduced circulating levels of adiponectin. Despite bigger adipocytes leptin expression was also downregulated in both depots at different reperfusion times; however, this did not lead to lower circulating leptin concentration (Figure 2B,C, Figure S8). The gene expression of lipogenic genes as *Srebf1* (Srebp1c) and the target gene of PPARγ *Dgat2* were downregulated in both depots, with stronger regulation again in iWAT (Figure 2B and Figure S8).

**FIGURE 3:**
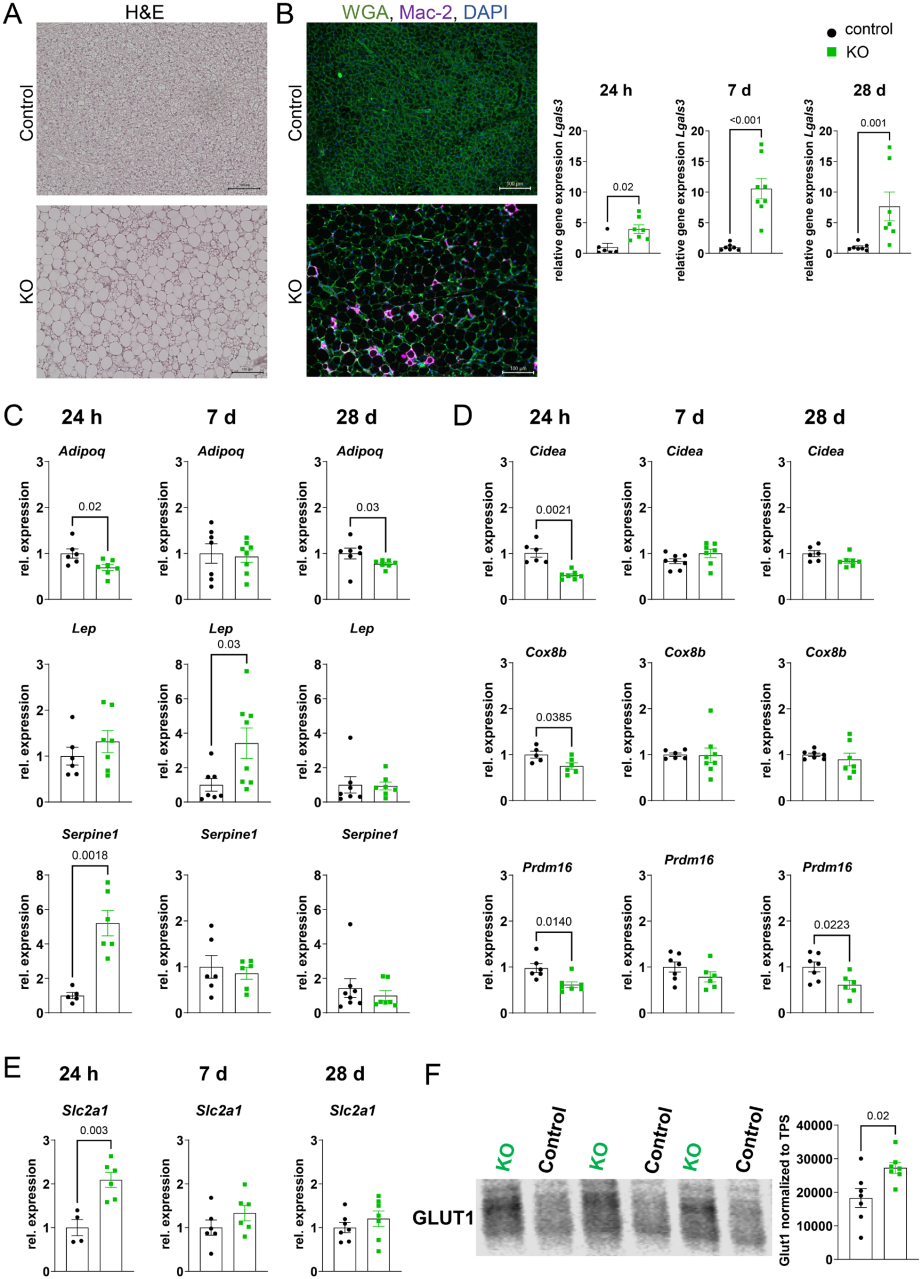
Phenotype of brown adipose tissue (BAT) in iatATGL‐KO mice after cardiac ischemia/reperfusion. (A) Exemplary images of H&E stained BAT of iatATGL KO and control mice after 28 days rep. (B) Exemplary images of immunofluorescence staining for Mac2 (purple) and WGA (green) in BAT of iatATGL KO and control mice after 7 days rep. Relative gene expression of Galectin 3 (*Lgals3*) in BAT after 24 h, 7 days and 28 days rep. *n* = 6–8, Mann–Whitney test. Relative gene expression of (C) adipokines adiponectin (*Adipoq*), leptin (*Lep*) and PAI‐1 (*Serpine1*) (D) browning markers *Cidea*, *Cox8b* and *Prdm16* and (E) *Slc2a1*. *n* = 6–8, unpaired two‐tailed *t*‐test, Welch's test, Mann–Whitney test. (F) Western blot analysis of GLUT1 protein expression in KO and control BAT after 24 h rep. Shown are exemplary blots and normalization to total protein stain (TPS). *n* = 7, unpaired two‐tailed *t*‐test. Full blots are shown in Figure S9B. All data are presented as mean ± SEM.

Next to white adipose tissue depots, the adiponectin Cre‐driver also leads to KO of ATGL in brown adipose tissue. This depot was therefore also highly altered by iatATGL‐KO, as already visible by eye (Figure S1A) and by histological analysis (Figure 3A), showing large and unilocular adipocytes, which are unusual for BAT. A higher number of nuclei was already observed in the H&E staining and hinted towards infiltrating cells, which were identified by subsequent immunofluorescence staining as Mac‐2 positive macrophages. These mainly surrounded unilocular adipocytes as crown‐like structures (Figure 3B). This striking finding was further supported by a strong upregulation of the macrophage marker galectin 3 (*Lgals3*) at all reperfusion time points. BAT is known for its high secretory activity of several batokines. We therefore analyzed the gene expression of different adipokines and found mainly adiponectin, leptin, and PAI‐1 (*Serpine1*) expression altered. In line with results from iWAT, adiponectin was downregulated (24 h and 28 days rep), while leptin was upregulated (7 days rep). Interestingly, also PAI‐1 expression was upregulated; however, this seemed to be a transient process, as this was only visible at 24 h rep (Figure 3C). We next analyzed several browning markers, which were mainly downregulated after 24 h rep (Figure 3D). This was in line with the finding from immunofluorescence staining for UCP1, which showed that the unilocular adipocytes lack UCP1 expression (Figure S9A). When analyzing the metabolic adaptation of BAT after I/R, we found an upregulation of GLUT1 at gene expression and protein level in the early reperfusion after cardiac ischemia, indicating that during this time, BAT ATGL might play an important role in regulating whole body energy homeostasis.

### 3.3 iatATGL‐KO BAT Exhibits Cardiac Ischemia‐Specific Disturbances

**FIGURE 4:**
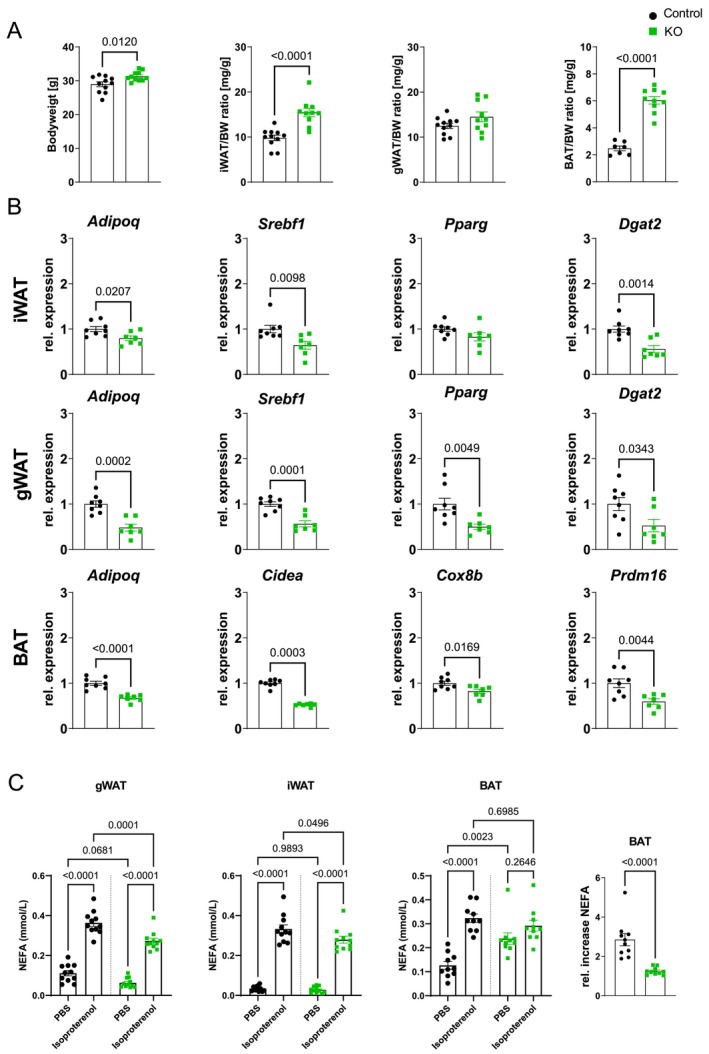
Adipose tissue biology at baseline. (A) Bodyweight, iWAT/BW‐ratio, gWAT/BW‐ratio and BAT/BW‐ratio from iatATGL‐KO and control mice at baseline. *n* = 7–12, unpaired, two‐tailed *t*‐test. (B) Relative gene expression of adiponectin (*Adipoq*), Srepb1c (*Srebf1*), *Pparg*, *Dgat2* in iWAT and gWAT and *Cidea, Cox8b* and *Prdm16* in BAT of KO relative to control. *n* = 7–8, unpaired, two‐tailed *t*‐test or Mann–Whitney test. (C) NEFA level released from iWAT, gWAT and BAT explants after 1 h incubation with PBS or Isoproterenol. Absolute NEFA levels in supernatant and relative increase of NEFA in BAT (Iso/PBS). *n* = 10–11, one‐way ANOVA with Tukey's multiple comparisons test or Mann–Whitney test. All data are presented as mean ± SEM.

To assess whether the alterations in adipose tissue biology were related to the iatATGL‐KO itself or to additional cardiac ischemia, the main observations were analyzed also in non‐operated animals. Different from post‐ischemic values, bodyweight was slightly increased, as well as iWAT and BAT to bodyweight ratios (Figure 4A). On gene expression level, especially adiponectin as well as the lipogenic gene profile was already reduced without cardiac ischemia in iWAT and gWAT. In BAT, the reduction in browning markers and increased inflammation were also observed at baseline, indicating a dysfunctional BAT due to ATGL‐KO (Figure 4B, Figure S10). Interestingly, *Slc2a1* and *Serpine1* gene expression were not altered at baseline (Figure S10A), indicating that the upregulation at 24 h rep is a specific metabolic adaptation of iatATGL‐KO BAT to cardiac I/R.

To further distinguish the contribution of the different depots, ATGL activity was assessed ex vivo in adipose tissue explants by stimulation with isoproterenol (Iso) and measurement of NEFA levels in the supernatant. In iWAT and gWAT, the released NEFAs were significantly lower in iatATGL‐KO samples compared to controls. Despite KO of ATGL, BAT showed higher NEFA levels in the vehicle (PBS)‐treated group compared to controls. However, the relative increase by Iso stimulation was significantly lower, showing again a markedly disturbed BAT function (Figure 4C).

### 3.4 Spatial Transcriptomics Analysis Reveals Niche‐Specific Regulation and Enhanced Cardiac Stress Response in iatATGL‐KO Hearts

**FIGURE 5:**
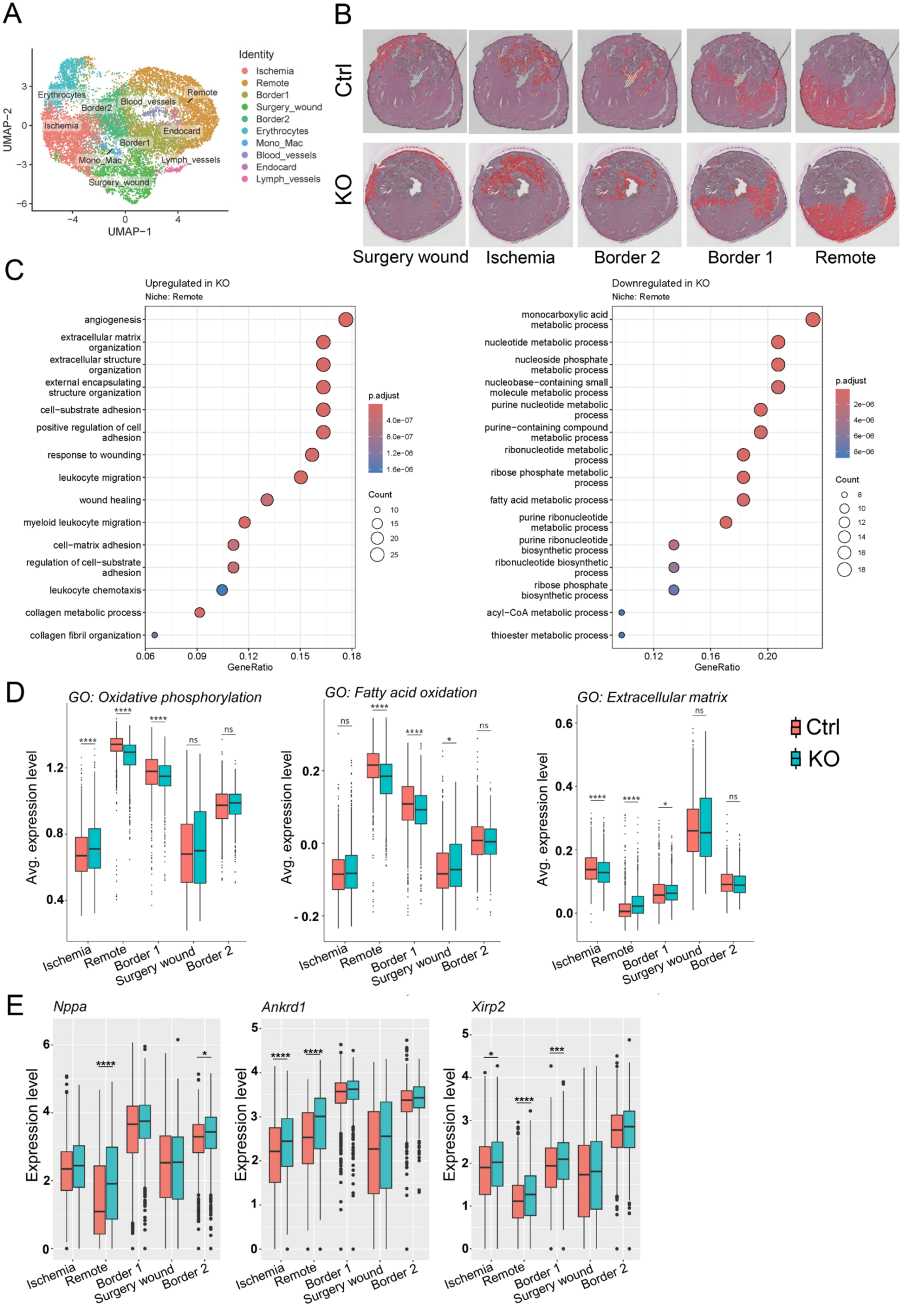
Spatial transcriptomic analysis of hearts of iatATGL‐KO and control mice after 24 h rep. (A) UMAP plot of niches in the heart after cardiac ischemia/reperfusion. (B) Spatial feature plot of indicated niches to visualize location in the heart. (C) Overrepresentation analysis of up‐ or downregulated genes in KO vs. control in the remote niche. (D) Expression level of gene signatures of interest derived from the gene ontology terms “oxidative phosphorylation” (GO:0006119), “fatty acid oxidation” (GO:0019395) and “extracellular matrix” (GO:0031012) in KO and control in the indicated niches. (E) Expression level of genes related to mechanical stress in KO and control in the indicated niches. *p < 0.05, **p < 0.01, ***p < 0.001, ****p < 0.0001.

To get insights into molecular alterations of iatATGL‐KO hearts, spatial transcriptomic analysis was performed in 3 KO and control hearts after 60 min ischemia and 24 h reperfusion. Integrated clustering revealed 5 main cardiac niches according to the zones arising after cardiac I/R, namely “remote”, “border 1”, “border 2”, “ischemia” and “surgery wound”. Additional 5 niches consisted of only a minor number of spots and could be identified according to their niche markers and localization as “blood vessels”, “lymph vessels”, “endocard”, “erythrocytes” and “monocytes/macrophages” (Figure 5A,B, Figure S11D). Differential gene expression showed that the remote zone exhibited the highest number of differentially regulated genes (Figure S11E). Overrepresentation analysis (ORA) of these genes revealed different metabolic processes as “monocarboxylic acid metabolic processes”, “nucleotide metabolic processes” and “fatty acid metabolic processes” to be downregulated in KO hearts. In line with this, also gene signatures arising from the gene ontology (GO)‐terms “oxidative phosphorylation” and “fatty acid oxidation” were significantly downregulated in remote and border 1 (Figure 5C,D).

Surprisingly, the ORA of upregulated genes in the remote zone resulted in terms regarding extracellular matrix organization. This went along with an upregulation of genes related to cardiac mechanical stress, such as *Nppa*, *Nppb*, *Ankrd1*, *Xirp2*, *Des*, and others (Figure 5E, Figure S12), indicating an enhanced mechanical stress within the remote zone of iatATGL‐KO hearts. Ingenuity pathway analysis (Qiagen) of remote zone DEGs revealed TGFB1 as the top upstream regulator and “Integrin cell surface interactions” and “Extracellular matrix organization” as canonical pathways with the highest positive z‐score (4.472 and 4.395) (Figure S13), which further supported mechanical stress and enhanced stretch within the remote zone of iatATGL‐KO hearts.

### 3.5 ATGL‐KO Hearts Exhibit Higher Basal Oxygen Consumption and Higher Dependency on Glucose as Substrate After 24 h I/R

**FIGURE 6:**
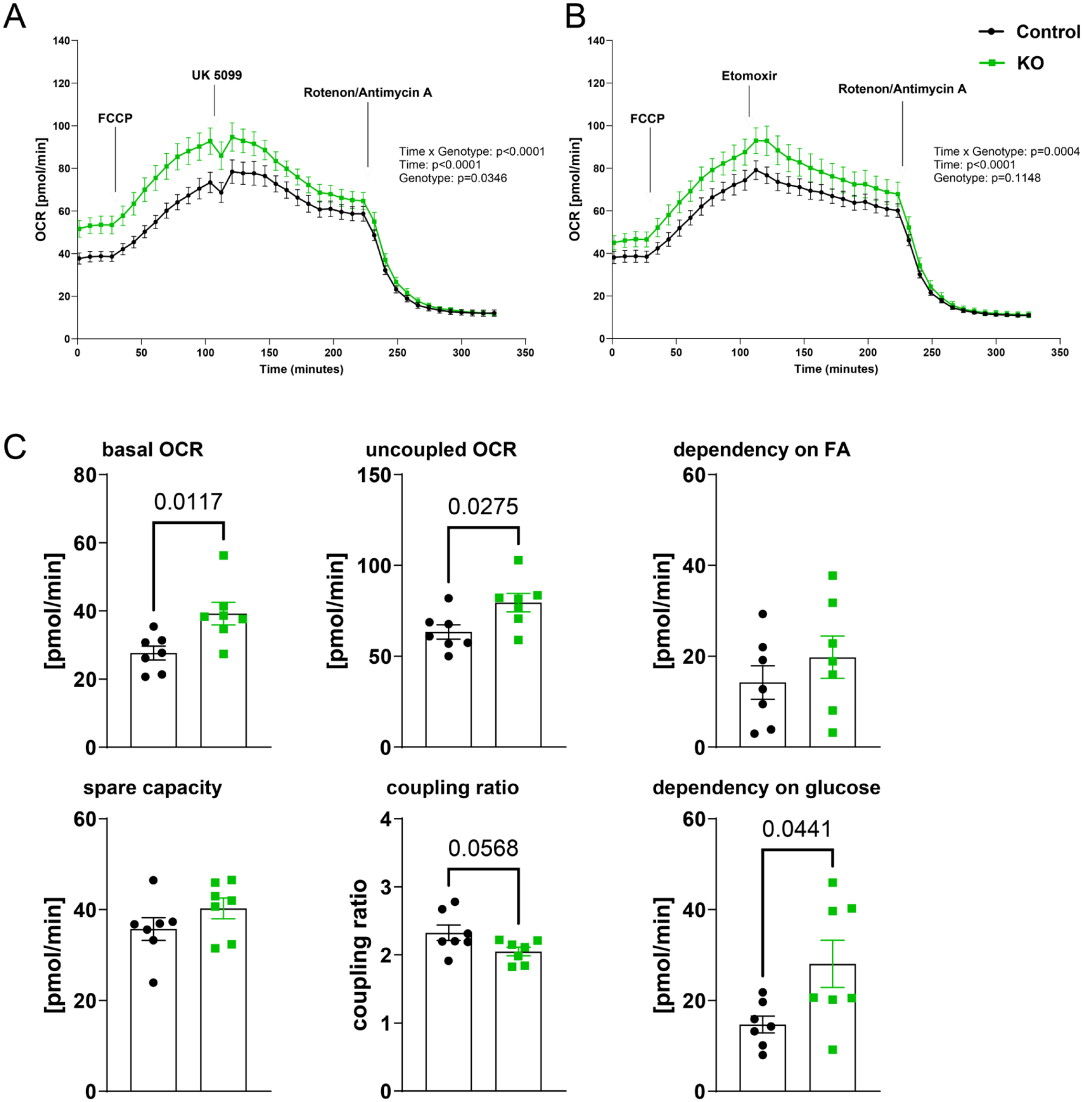
Extracellular flux measurements in remote area tissue from iatATGL‐KO and control hearts after 24 h rep. (A, B) Oxygen consumption rate (OCR) traces in KO and control tissue throughout the experiment under treatment with indicated inhibitors. Treatment with UK5099 indicates dependency on glucose (A), treatment with etomoxir indicates dependency on fatty acids (B). *n* = 7, two‐way ANOVA. (C) basal and uncoupled OCR, spare capacity and coupling ratio, dependency on glucose and fatty acids (FA) in KO and control remote tissue. *n* = 7, unpaired two‐tailed *t*‐test, Welch's test. All data are presented as mean ± SEM.

To assess alterations in cardiac substrate metabolism due to iatATGL‐KO, we performed extracellular flux measurements in cardiac tissue slices from the remote area of ATGL‐KO and control hearts, namely the septum, after 24 h rep. The specific inhibitors etomoxir (CPT‐inhibitor) and UK 5099 (MPC‐inhibitor) were used to assess the contribution of either palmitate or glucose to uncoupled oxygen consumption. Figure 6A,B show traces of oxygen consumption rate at baseline, after uncoupling via FCCP and inhibition with either UK 5099 (Figure 6A) or etomoxir (Figure 6B) for control and KO tissue. To measure the non‐mitochondrial contribution, rotenone and antimycin A were added at the end to each experiment. As already visible in the traces, the basal and uncoupled mitochondrial OCR were higher in the remote area of hearts from iatATGL‐KO mice (Figure 6C). The coupling ratio, however, strongly trended to be lower, pointing to a lower potential of hearts from iatATGL‐KO mice to maximize their oxygen consumption. Interestingly, etomoxir did not alter the reduction in OCR in iatATGL‐KO samples compared to control, while UK 5099 had a stronger effect on lowering uncoupled OCR in remote myocardium from iatATGL‐KO hearts. This indicates a higher dependency of the remote myocardium of iatATGL‐KO animals on glucose as substrate after 24 h rep. Interestingly, none of these differences were observed in iatATGL‐KO hearts without cardiac ischemia (Figure S14), indicating a specific role of adipocyte ATGL in cardiac metabolism after cardiac I/R.

## 4 Discussion

The activation of peripheral lipolysis after myocardial infarction, mainly due to an increase in circulating catecholamines, has been a well‐known pathophysiological reaction leading to elevated levels of free fatty acids in the circulation (Essop and Opie 2020). The discovery of ATGL as the first and rate‐limiting enzyme in the lipolytic cascade 20 years ago (Zimmermann et al. 2004; Villena et al. 2004; Jenkins et al. 2004; Haemmerle et al. 2006) led to a variety of studies in the field of heart—adipose tissue crosstalk in the context of heart failure (Parajuli et al. 2018; Salatzki et al. 2018; Takahara et al. 2021; Thiele et al. 2021). In our work, we could show before that inhibition of murine ATGL using the small‐molecule inhibitor Atglistatin prior to cardiac ischemia improved remote myocardium function 7d post I/R (Bottermann et al. 2020). Additionally, our analysis of post‐I/R alterations in murine white adipose tissue depots revealed, next to the known acute lipolytic activation in response to cardiac I/R, also a more chronic activation of the inguinal WAT depot, including an upregulation of ATGL (Wang et al. 2022). In the current project we therefore investigated the role of adipocyte ATGL in post‐ischemic cardiac remodeling in more detail by using tamoxifen‐inducible adipocyte specific ATGL‐KO mice, which underwent cardiac ischemia and reperfusion. An advantage of the inducible system over constitutive KO models is less fat accumulation and metabolic adaptation during development, helping to focus on the effects of ATGL during and after cardiac I/R.

Surprisingly, the iatATGL‐KO mice showed an aggravated cardiac dysfunction after cardiac I/R as well as an enlarged scar size. In adipose tissue, this phenotype went along with strong alterations mainly in iWAT and BAT, including enlarged adipocytes, inflammation, reduced lipogenesis, and reduced levels of circulating adiponectin.

It is well known that adipocyte specific KO of ATGL alters different adipose tissue depots as BAT and WAT; however, we report here for the first time the effects in an inducible, adipocyte‐specific KO model. Even before cardiac ischemia, a slight increase in body weight, iWAT/BW‐ and BAT/BW‐ratio was observed. In line with other reports (Wei Wu et al. 2012; Schoiswohl et al. 2015), adipocyte size in gWAT was unchanged and gWAT/BW ratio only increased at early reperfusion time points, a finding which might be attributed to shrinking adipocytes in gWAT shortly after cardiac ischemia and which was not due to fading of the KO with time after induction (Figure S6). Surprisingly, this was nevertheless accompanied by a reduction in the lipogenic gene expression profile at baseline and partly after I/R. In iWAT, the effects on adipocyte size and lipogenic gene profile were more pronounced after cardiac I/R; thus, our data show that iWAT of iatATGL‐KO mice is more affected after myocardial infarction than gWAT, which is in line with our findings that iWAT is in general more prone to alterations after I/R and *Pnpla2* expression is upregulated after I/R in iWAT (Wang et al. 2022).

Even more pronounced than the increase in iWAT mass, BAT weight and adipocyte size were enlarged and brown adipocytes acquired a white‐like, unilocular phenotype, a phenomenon which was also reported before (Ahmadian et al. 2011; Schreiber et al. 2017). This strong phenotype in BAT went along with macrophage infiltration, loss of UCP1 in unilocular adipocytes and reduction in BAT activation markers, such as *Cidea*, *Cox8b* and *Prdm16*, with and without cardiac ischemia. BAT is known to be activated after MI (Martí‐Pàmies et al. 2023), a mechanism which seems to be disturbed in iatATGL‐KO BAT. The activation likely increases energetic needs, but since stimulation of intracellular lipolysis is diminished in iatATGL‐KO BAT, other substrates are needed to meet the requirements. Accordingly, the insulin‐independent glucose transporter 1 (Glut1, *Slc2a1*) was upregulated in KO‐BAT specifically after 24 h I/R. In addition, whitening of brown adipocytes triggers inflammation and brown adipocyte death (Kotzbeck et al. 2018), which may diminish the protective role of BAT activation after MI and thereby yield an explanation for the aggravated cardiac dysfunction after I/R in iatATGL‐KO mice.

All depots, BAT, iWAT and gWAT are also a source of circulating factors such as NEFAs, adipokines and batokines. Consistently with pertinent literature, the elevation in circulating NEFA levels shortly after cardiac ischemia was blunted in iatATGL‐KO mice, an effect which is mainly considered as cardioprotective (Grossman et al. 2013) since NEFAs have negative regulatory effects on glucose oxidation (Randle et al. 1963), exert pro‐arrhythmogenic functions (Kurien et al. 1971), induce lipotoxicity (D'Souza et al. 2016; Perman et al. 2011) and uncouple mitochondria (Borst et al. 1962). According to our ex vivo analyses, this effect is mainly attributed to reduced NEFA release from iWAT and gWAT. In addition, gene expression and multiplex analysis also revealed that adipokines were affected in iatATGL‐KO mice, as adiponectin was reduced at the gene expression level as well as in circulation. Adiponectin is secreted by healthy adipose tissue when tissue weight is low and is reduced after myocardial infarction (Kojima et al. 2003). Adiponectin is considered an anti‐inflammatory and cardioprotective adipokine (Essick et al. 2011; Shibata et al. 2007) and its reduction in iatATGL‐KO mice may represent a further mechanism for worse cardiac outcome after I/R. One of the major transcriptional regulators of adiponectin is PPARy (Fang and Judd 2018), a transcription factor which expression was partly reduced itself. Even more pronounced was the downregulation of its downstream target *Dgat2*, indicating a reduced PPARγ activity in iatATGL‐KO after I/R, especially in iWAT.

In BAT PAI‐1 (*Serpine1*) was upregulated early after reperfusion, while its expression was unchanged at baseline and at later reperfusion timepoints. PAI‐1 is considered a profibrotic factor, which enhances cardiac fibrosis after myocardial infarction (Takeshita et al. 2004; Zaman et al. 2009). In our spatial transcriptomics data, we find an upregulation of *Serpine 1* in the remote and border 1‐zone (Figure S12) where it could contribute to the fibrotic remodeling after I/R by inhibiting matrix metalloproteinases (Ghosh and Vaughan 2012).

Taken together, adipose‐tissue derived factors are altered time‐ and depot‐specific in iatATGL‐KO animals and may therefore have divergent effects on cardiac remodeling. While reduction in NEFAs is rather cardioprotective, reduction in adiponectin and increased PAI‐1 expression support adverse cardiac remodeling. Given the fact that NEFAs are only elevated transiently after MI while the effects of adipokines last throughout the whole remodeling process, the observed rather mild reduction in NEFAs and their protective effect might be overruled by adverse signaling via adipokines.

Due to the fact that several alterations especially in BAT occurred mainly in the early reperfusion phase, we focused our investigation in cardiac tissue on the 24 h rep timepoint. Cardiac ischemia/reperfusion results in a highly heterogeneous heart with different zones as ischemic, border and remote zone with different cellular compositions and which undergo strong transitions through several phases in the process of wound healing. To assess the regional differences within the heart after MI on the transcriptional level, we performed spatial transcriptomic analysis at an anatomical level in the left ventricle where both remote and ischemic areas are visible in one cross section. Cluster analysis of transcriptionally similar regions identified five cardiac niches (non‐ischemic remote area, ischemic area, two border zone areas, surgery wound from the experimental infarct induction), from which the expressing niche‐markers were in line with previous reports (Calcagno et al. 2022; Kuppe et al. 2022; Yamada et al. 2022). In addition to the main cardiac niches, five more cell type specific niches were also identified by cluster analysis. Due to the limited number of spots they were composed of, and the fact that spatial transcriptomics does not yield a single cell resolution, these niches were not included in the deeper analysis. Differential gene expression analysis, overrepresentation analysis and ingenuity pathway analysis (Qiagen) in the remote niche identified a downregulation of different metabolic processes and surprisingly an upregulation of terms related to extracellular matrix and integrin signaling. In line with this, several mechanosensitive genes and cardiomyocyte stress markers were upregulated indicating an elevated mechanical stress in the remote myocardium of iatATGL‐KO mice, which is however at this timepoint not due to bigger ischemic injury. TGFβ was furthermore identified as highly predicted underlying regulator.

To further investigate the functional relevance of alterations in gene expression, we performed extracellular flux measurements with an established protocol (Bottermann et al. 2023), yielding information on cardiac substrate dependency. Given the fact that lipolysis is inhibited in iatATGL‐KO mice and circulating NEFA levels limited, it is not surprising that the hearts of iatATGL‐KO animals are more dependent on glucose as a substrate after 24 h rep. A similar effect was shown in mice with adipocyte‐specific KO of the ATGL co‐factor CGI‐58, which had an elevated cardiac glucose uptake, especially in stress situations, in this case cold stress (Choi et al. 2021). A second interesting observation was an elevated basal and uncoupled OCR in iatATGL KO hearts after 24 h rep, while the coupling ratio was close to significantly reduced. A higher basal OCR fits with the observation of upregulated mechanosensitive genes in remote and border zone, indicating elevated mechanical stress, which may lead to increased oxygen consumption as oxygen consumption correlates with wall tension (Kissling 1992; Gutterman and Cowley 2006). The reduced coupling ratio however indicates a limitation in the ability to maximize oxygen consumption, which fits with the downregulated signatures “fatty acid oxidation” and “oxidative phosphorylation” found in spatial transcriptomic analysis. Additionally, the enhanced mechanical stress might activate resident fibroblasts (Shah et al. 2021), which may contribute to the larger scar after I/R.

## 5 Conclusion

Taken together, adipocyte specific ATGL, mainly originating from inguinal WAT and BAT, is crucially involved in the cardiac remodeling process after cardiac ischemia and reperfusion. This is driven multifactorial most likely by reduced levels of adiponectin, reduced protective BAT function and increased mechanical stress in the remote and border zone of the heart, leading to elevated oxygen consumption, enhanced remodeling and subsequently aggravated cardiac dysfunction.

## Author Contributions

Conceptualization: H.Z., K.B.; Methodology: H.Z., S.G., L.W., A.H., S.L., T.L., K.B.; Investigation: H.Z., A.U., L.W., R.K., S.G., S.L., T.L., K.B.; Formal analysis: D.G., M.B., T.L.; Writing – original draft preparation: H.Z., K.B.; Writing – review and editing: H.Z., K.B., S.G., L.W., J.W.F., A.H., A.G.; Supervision: K.B., A.H., J.W.F., A.G.; Funding Acquisition: J.W.F., K.B.

## Funding

This work was supported in part by a grant of the Research Committee of the Medical Faculty of the University Hospital Düsseldorf to K.B., and by grants of the German Research Foundation CRC 1116, TPA08, and S01 to J.W.F. and IRTG 3109, P7 to K.B. and J.W.F.

## Ethics Statement

All animal experiments were performed according to national guidelines and approved by the authorities (LANUV, NRW).

## Conflicts of Interest

The authors declare no conflicts of interest.

## Supporting information


**Data S1:** cph470106‐sup‐0001‐Figures.pdf.

## Data Availability

Raw sequencing data of spatial transcriptomics experiments are available on EMBL‐EBI Biostudies (https://www.ebi.ac.uk/biostudies/) under accession number E‐MTAB‐12700. The code of the bioinformatic analysis can be accessed via https://doi.org/10.5281/zenodo.18346932.
