# Supplementary material for: ATGL From iWAT and BAT Is Crucial for Cardiac Remodeling and Metabolism After Myocardial Ischemia/Reperfusion
Source: Compr Physiol. 2026 Feb 4;16(1):e70106. doi: 10.1002/cph4.70106 (PMC12872207; doi:10.1002/cph4.70106)
Supplement: Supplementary file 1 — Data S1: cph470106‐sup‐0001‐Figures.pdf. [file CPH4-16-e70106-s001.pdf]

ATGL From iWAT and BAT Is Crucial for Cardiac Remodeling and Metabolism After Myocardial Ischemia/Reperfusion

Heba Zabri[1], Alisa Ucar[1], Luzhou Wang[1], Simone Gorreßen[1], Richard Kretschmer[1], Daniel Gorski[1], Tobias Lautwein[2], Mirela Balan[3], Stefan Lehr[4,5], Andre Heinen[6], Axel Gödecke[6,7], Jens W. Fischer[1,7], Katharina Bottermann[1]

[1] Institute for Pharmacology, Medical Faculty and University Hospital Düsseldorf, Heinrich Heine University Düsseldorf, Germany
[2] Genomics & Transcriptomics Laboratory (GTL) of the Heinrich-Heine University Düsseldorf, Germany
[3] Core Unit Bioinformatics (CUBI), Medical Faculty and University Hospital Düsseldorf, Heinrich Heine University Düsseldorf, Germany
[4] Institute for Clinical Biochemistry and Pathobiochemistry, German Diabetes Center (DDZ), Leibniz Center for Diabetes Research at Heinrich Heine University Düsseldorf
[5] German Center for Diabetes Research (DZD e.V.), Munchen-Neuherberg, Germany
[6] Institute for Cardiovascular Physiology, Medical Faculty and University Hospital Düsseldorf, Heinrich Heine University Düsseldorf, Germany
[7] CARID-Cardiovascular Research Institute Düsseldorf, Medical Faculty and University Hospital Düsseldorf, Heinrich Heine University Düsseldorf, Germany

Supporting Information

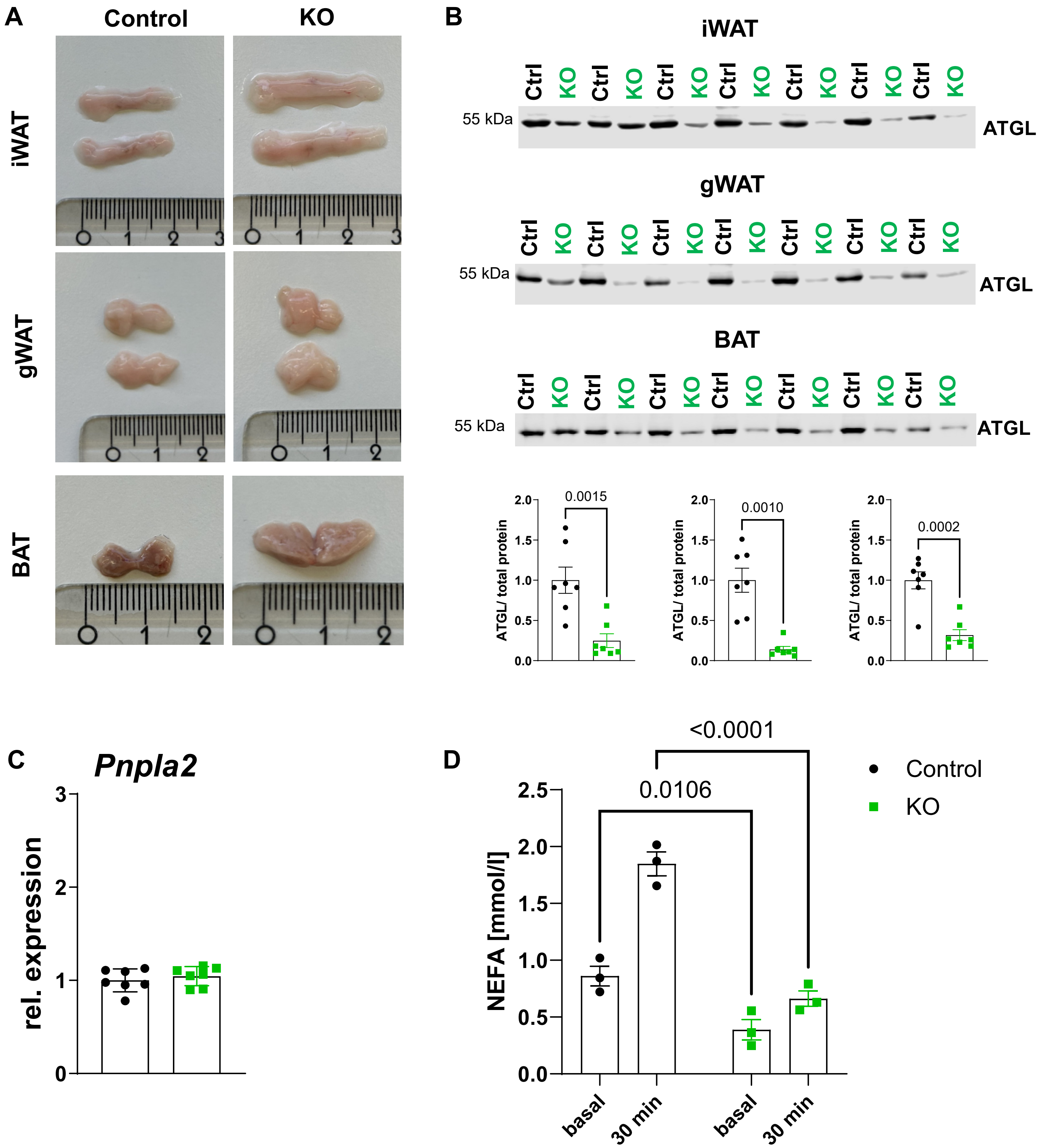

Fig. S1: Verification of ATGL-KO in different fat depots. A) Macroscopic images of iWAT, gWAT and BAT of control and KO animals B) Western blot analysis for ATGL-expression in iWAT, gWAT and BAT. Expression was normalized to total protein stain und relative to control. n=7.C) Relative gene expression of *Pnpla2* (ATGL) in cardiac tissue. n=7 D) NEFA level in control and KO serum before and after injection of β_3-agonist CL316,243. All Data are mean ± SEM, unpaired two-tailed t-test, Welch‘s test or two-way ANOVA with Sidak‘s multiple comparison test.

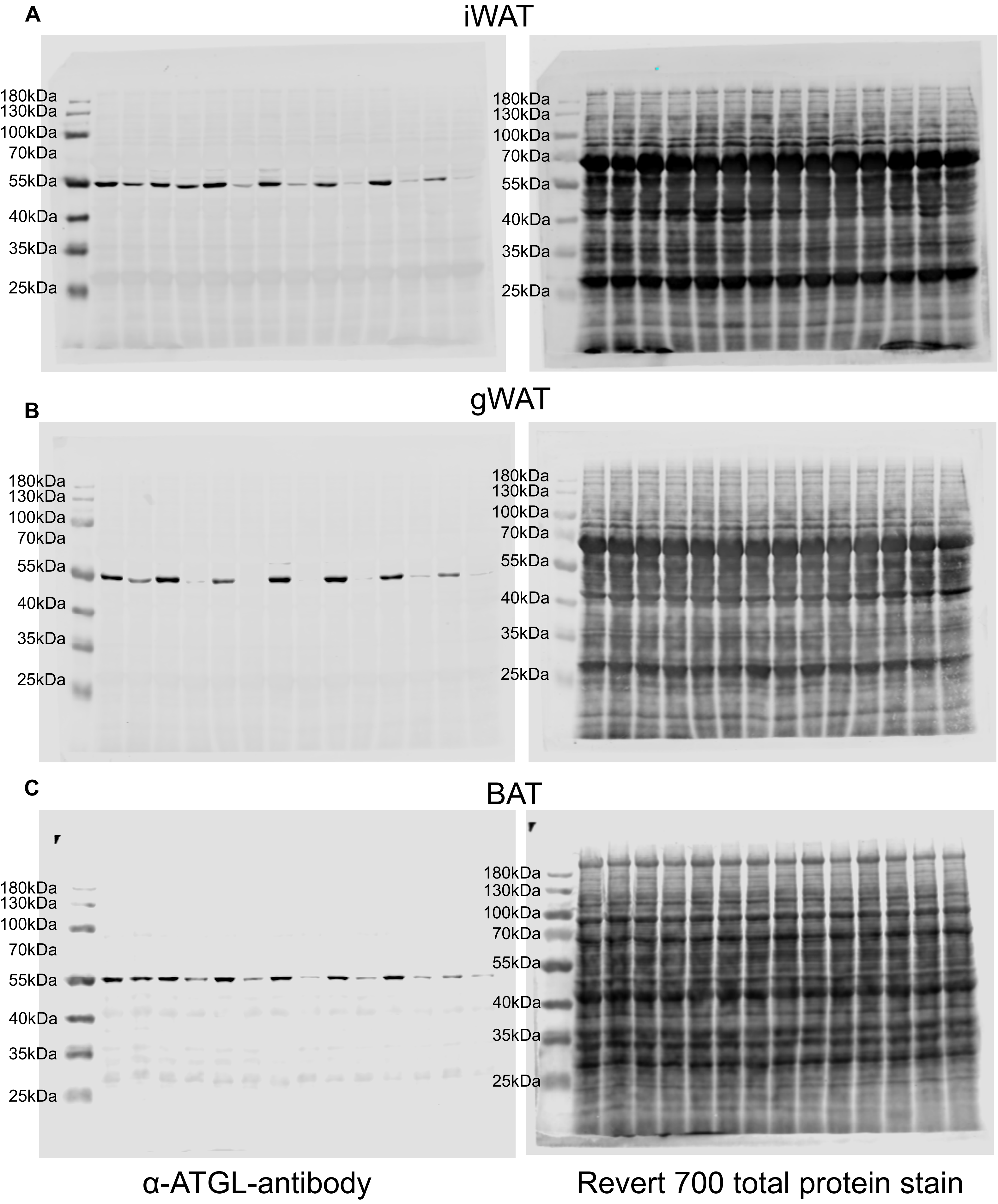

Fig. S2: Full size blots for Supplemental Fig. 1B and corresponding total protein stainings for iWAT (A), gWAT (B) and BAT (C).

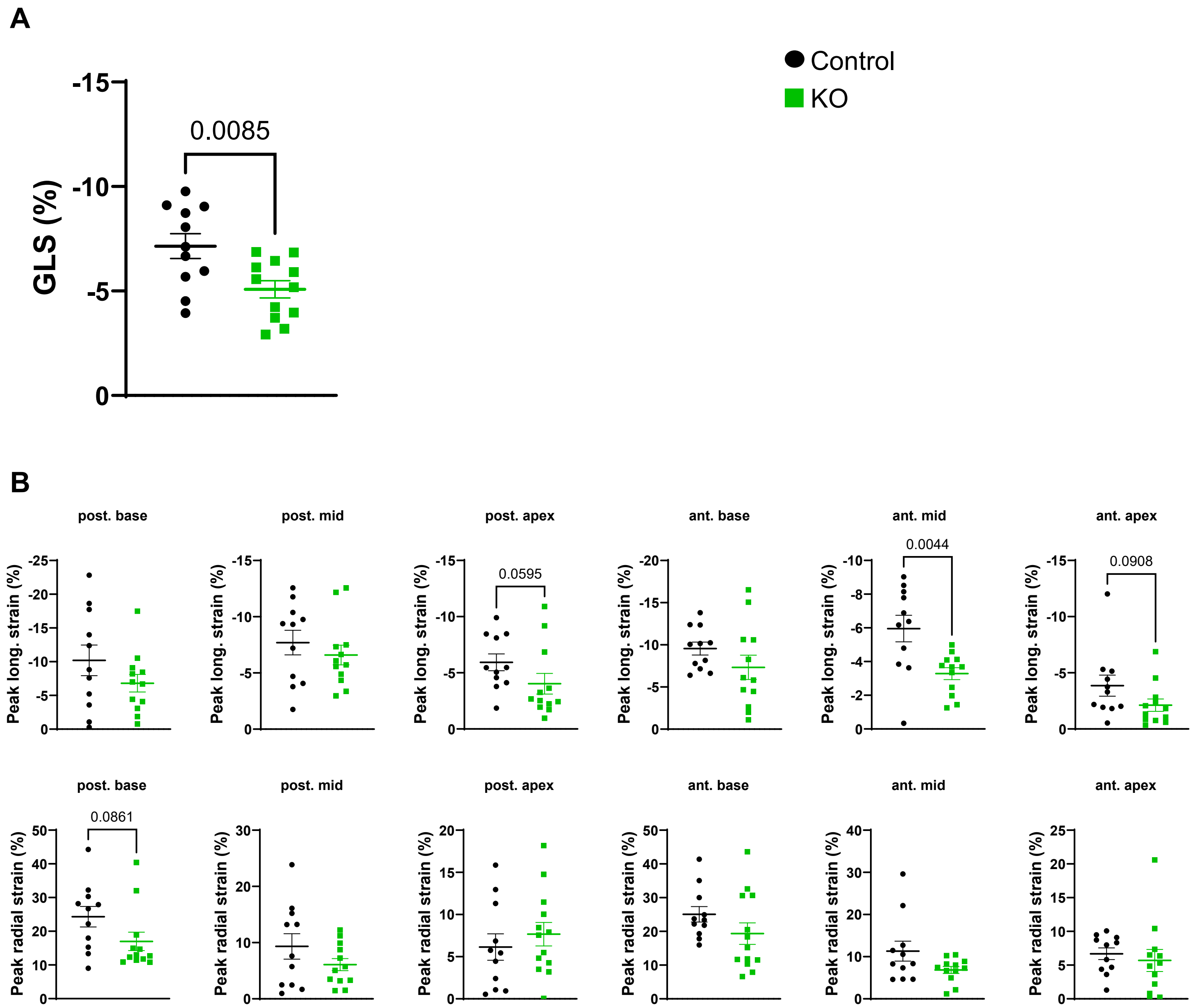

Fig. S3: Strain analysis of parasternal long axis echo data after 28 d I/R. A) Global longitudinal strain B) Peak longitudinal strain and peak radial strain for 6 segments of the heart: posterior base, mid and apex, anterior base, mid and apex. n=11-12, data are mean ± SEM, unpaired two-tailed t-test, Welch‘s test or Mann-Whitney test.

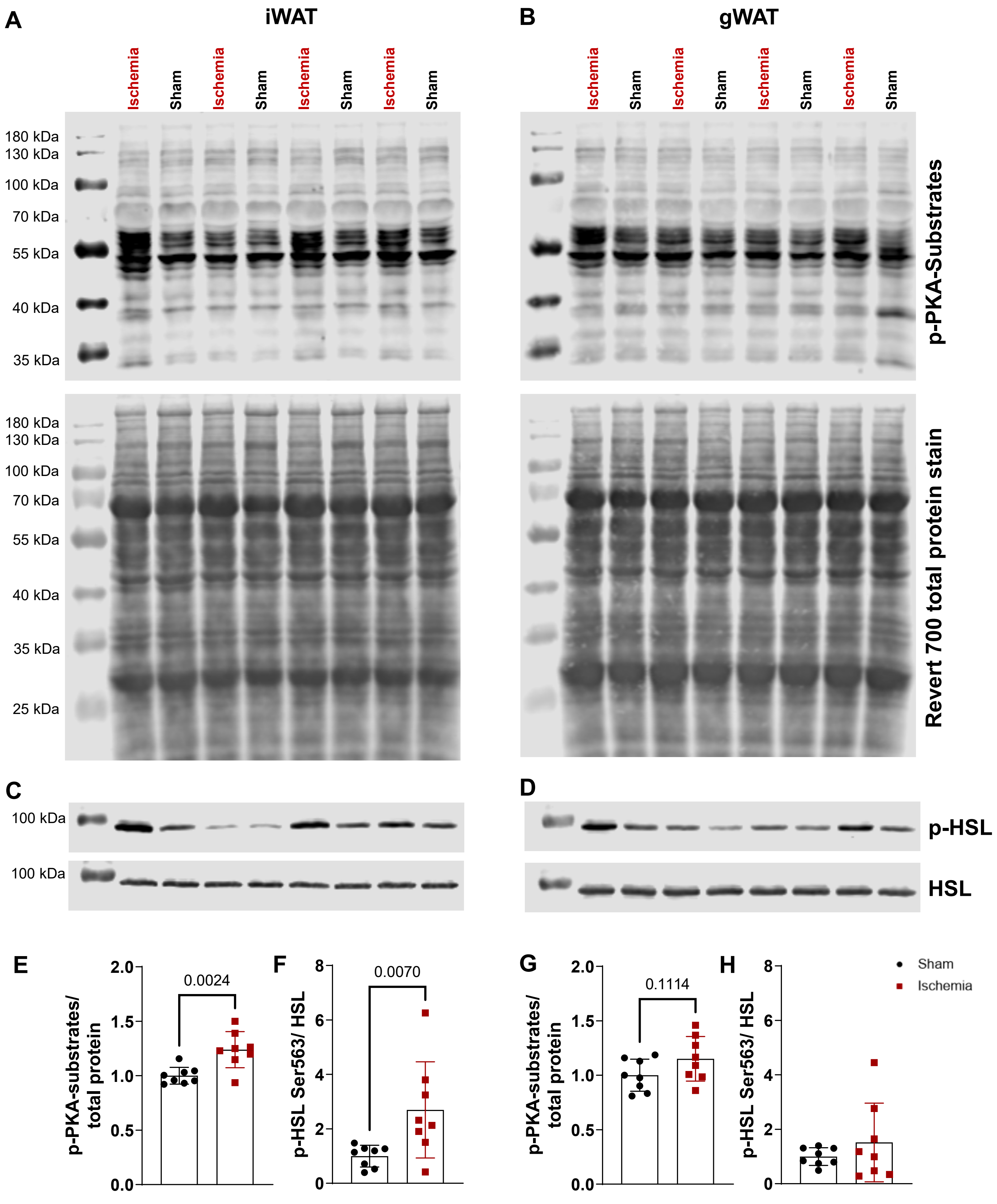

Fig. S4: Western blot analysis of iWAT (A, C) and gWAT (B, D) in sham and ischemia-operated C57Bl/6J male mice after 30 min reperfusion for p-PKA substrates and p-HSL Ser563. Signals were normalized to total protein staining or to total HSL. Quantification normalized to sham-group in iWAT (E,F) and gWAT (G,H). Data are mean ± SEM, Mann-Whitney or unpaired two-tailed t-test, n=8.

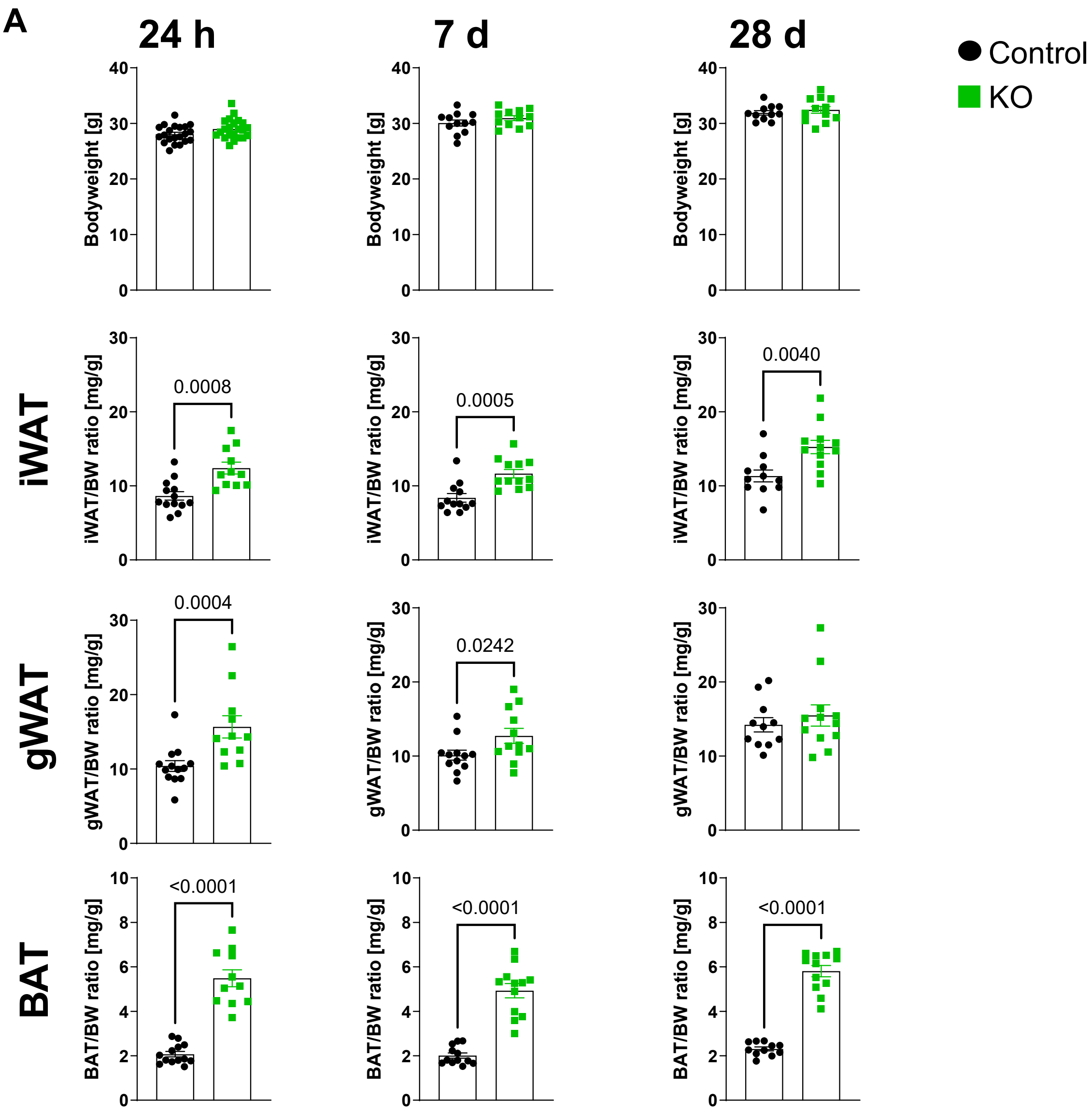

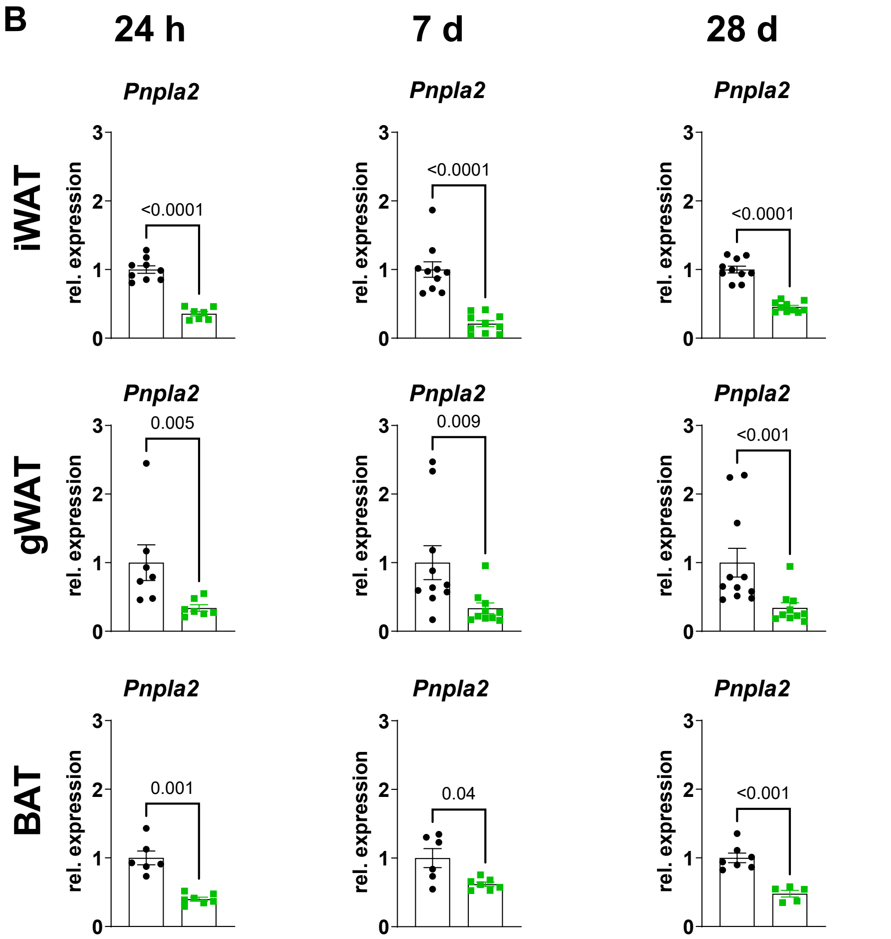

Fig. S5: Body and organweight. A) Bodyweight (BW) and gWAT, iWAT and BAT/BW ratio at all three reperfusion timepoints. n=11-22 B) Gene expression of Pnpla2 (ATGL gene) in all three depots and at all three reperfusion time points. n=5-11
All data are mean ± SEM, unpaired two-tailed t-test, Welch‘s test or Mann-Whitney test.

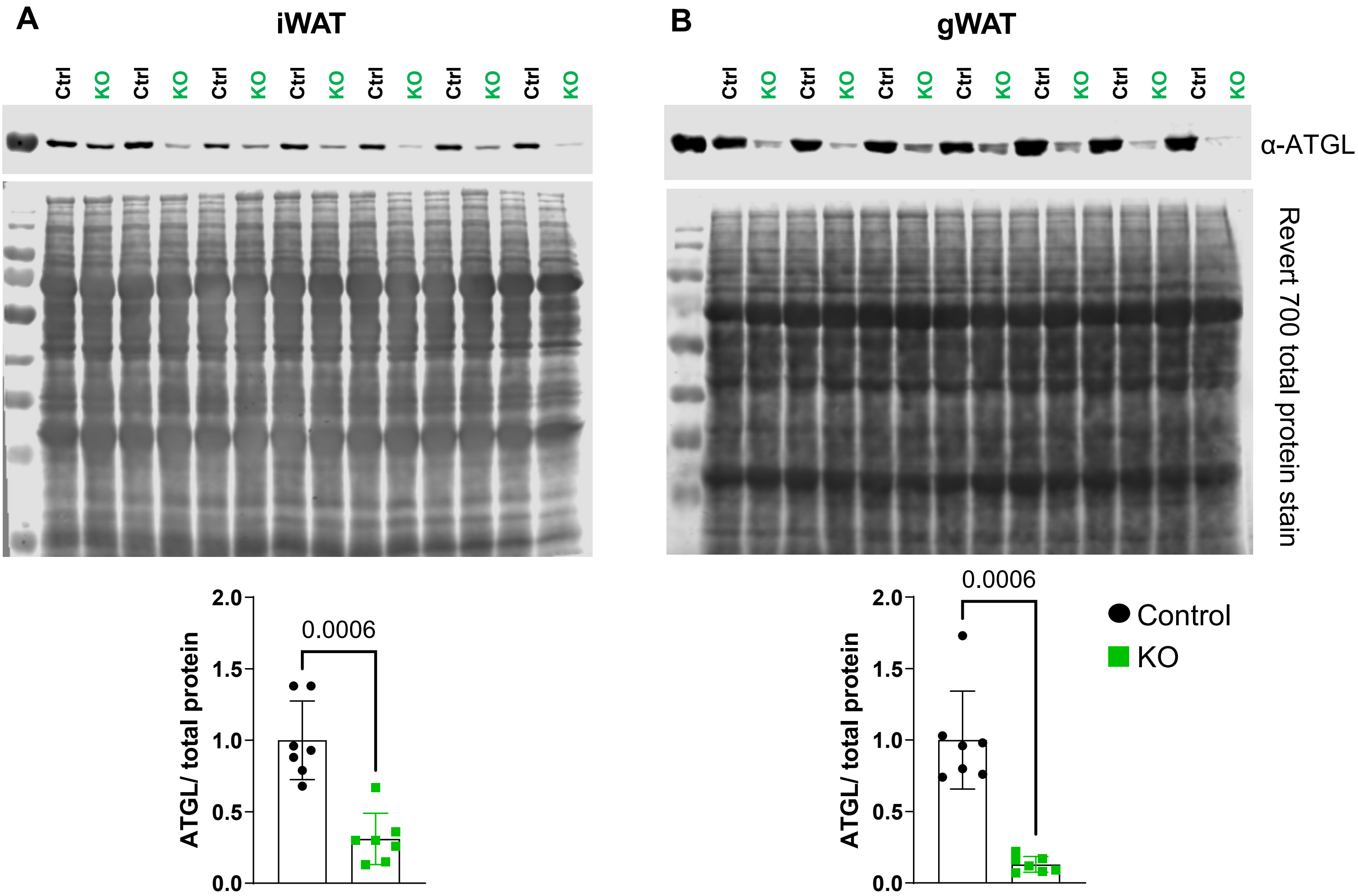

Fig. S6: Western blot analysis of iWAT (A) and gWAT (B) for expression of ATGL at the end of the experiment (28 d rep). Signals were normalized to total protein staining and quantified relative to control. Data are mean ± SEM, unpaired two-tailed t-test or Mann-Whitney test, n=7.

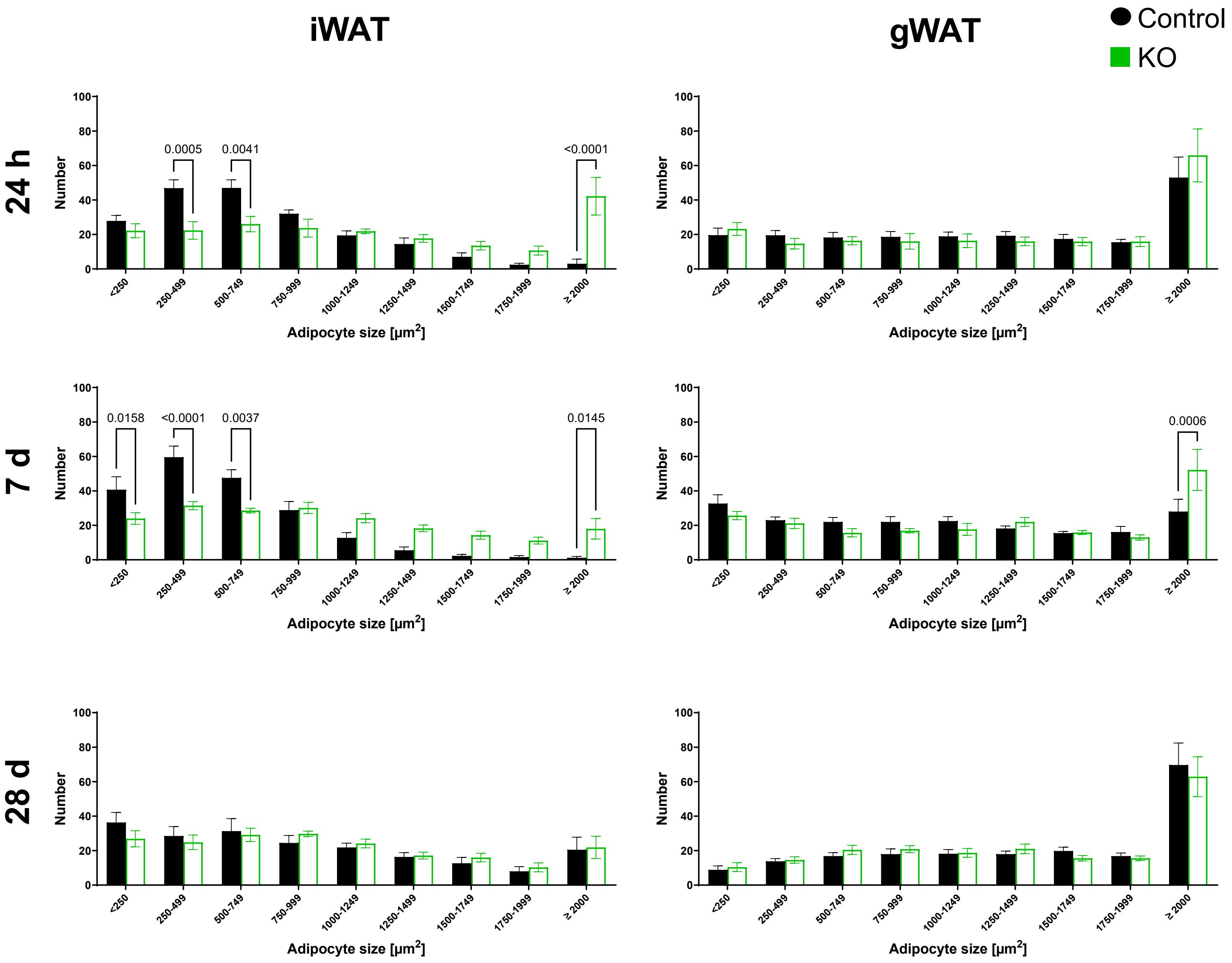

Fig. S7: Distribution of adipocyte size in iWAT and gWAT at all reperfusion time points. Data are mean ± SEM, two-way ANOVA with Sidak‘s multiple comparisons test, n=6-8.

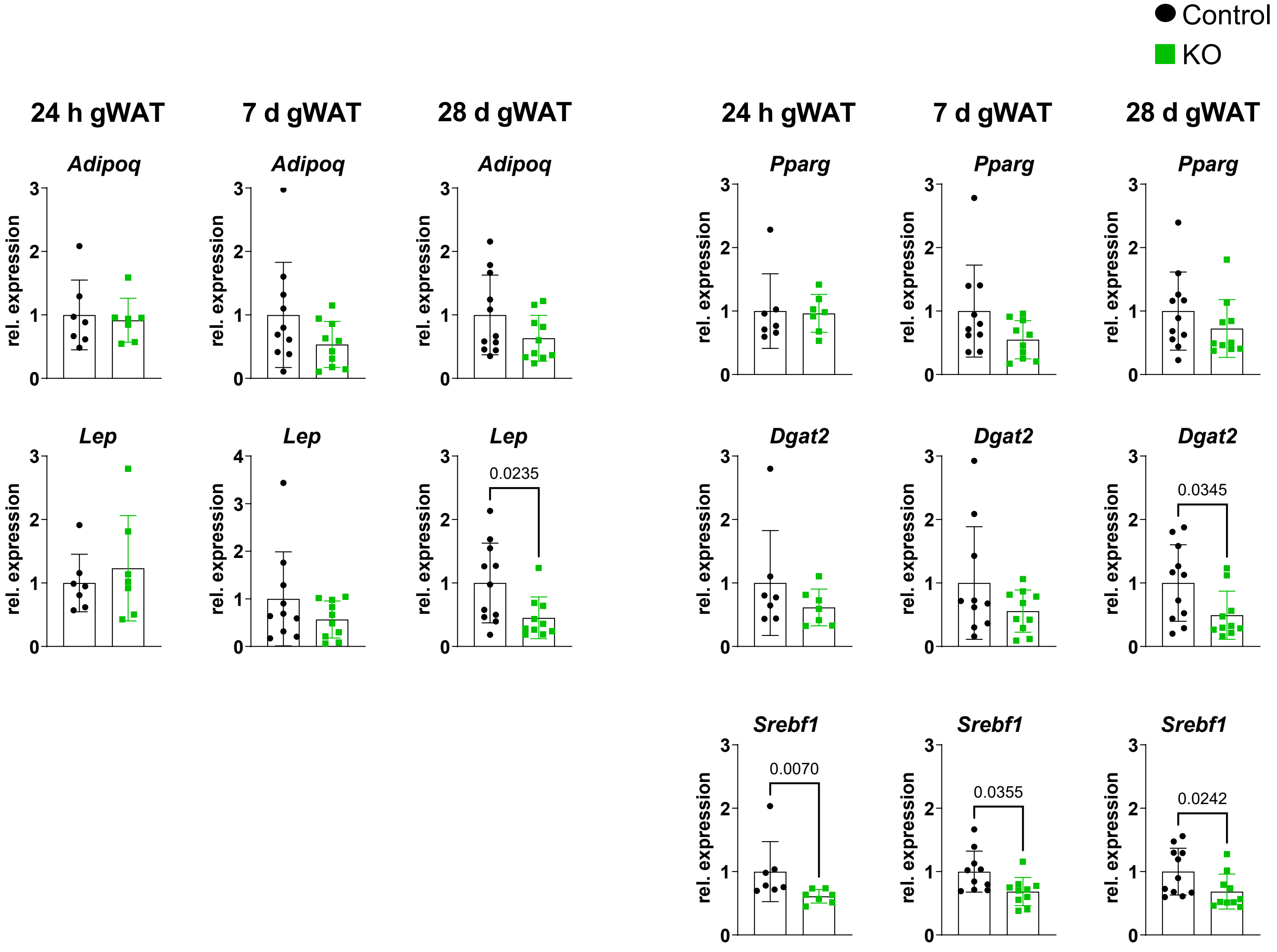

Fig. S8: Gene expression of adiponectin (*Adipoq*), leptin (*Lep*), *Pparγ*, *Dgat2* and Srebp1c (*Srebf1*) in gWAT of KO relative to control after 60 min ischemia and either 24 h, 7 d or 28 d rep. Data are mean ± SEM, unpaired two-tailed t-test, n=7-11.

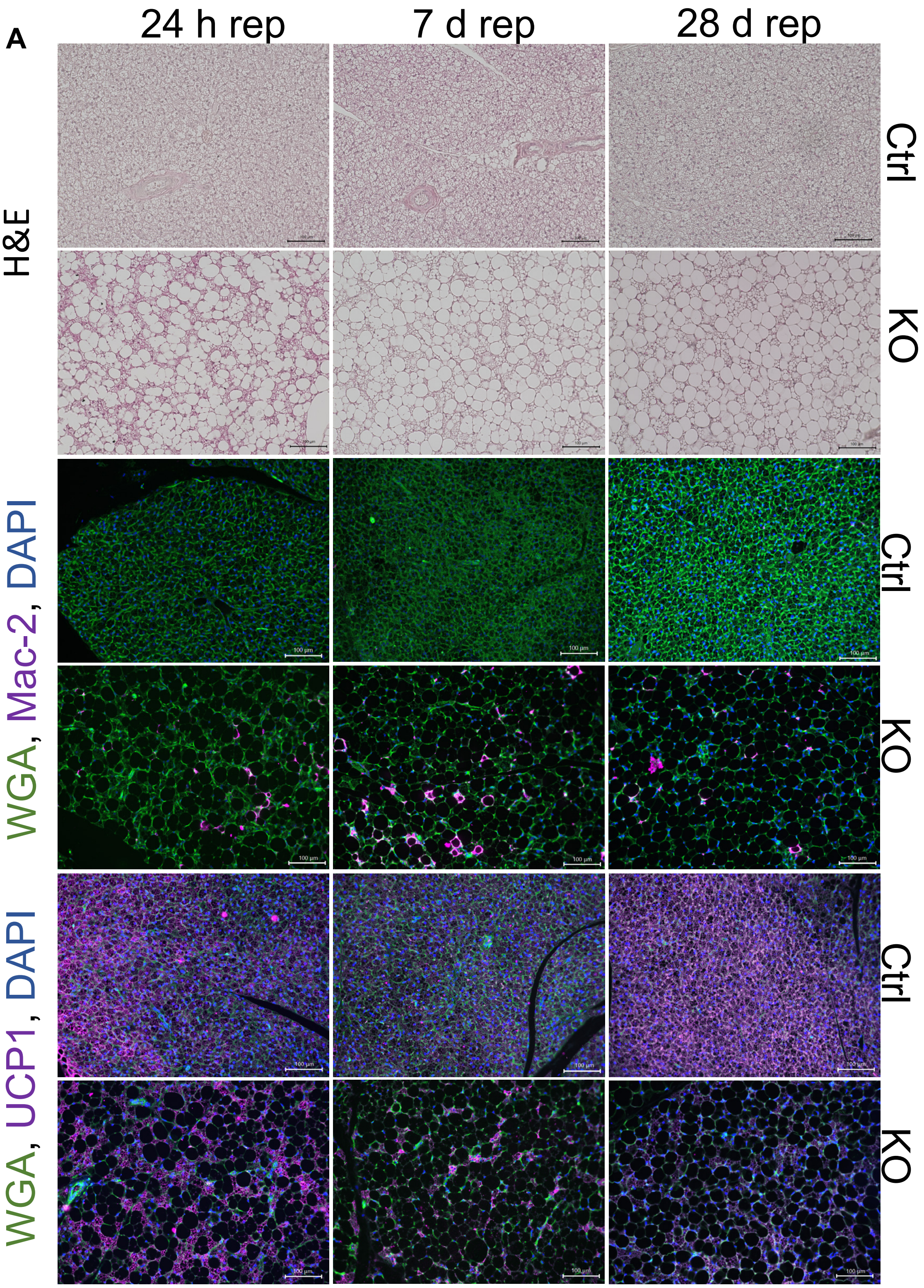

Fig. S9: A) Histological analysis of BAT at all reperfusion time points. B) Full size western blot images of Fig. 3F. A-Glut1-antibody left, Revert 700 total protein stain right.

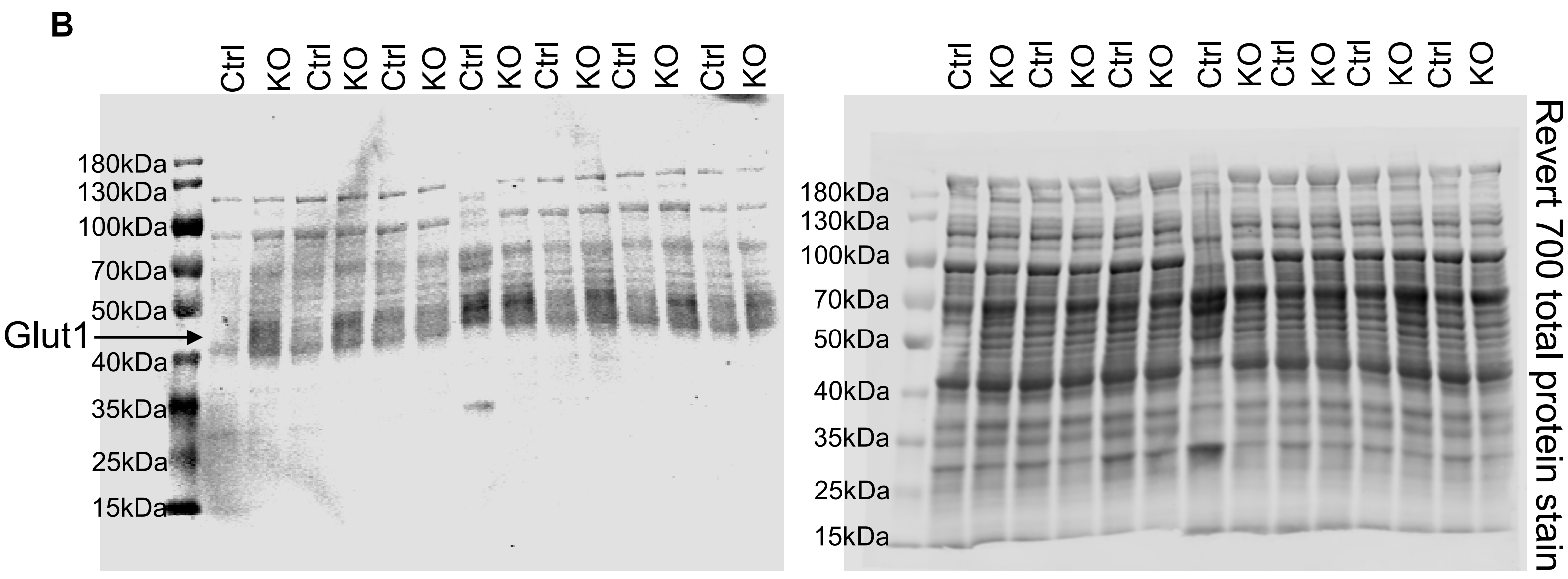

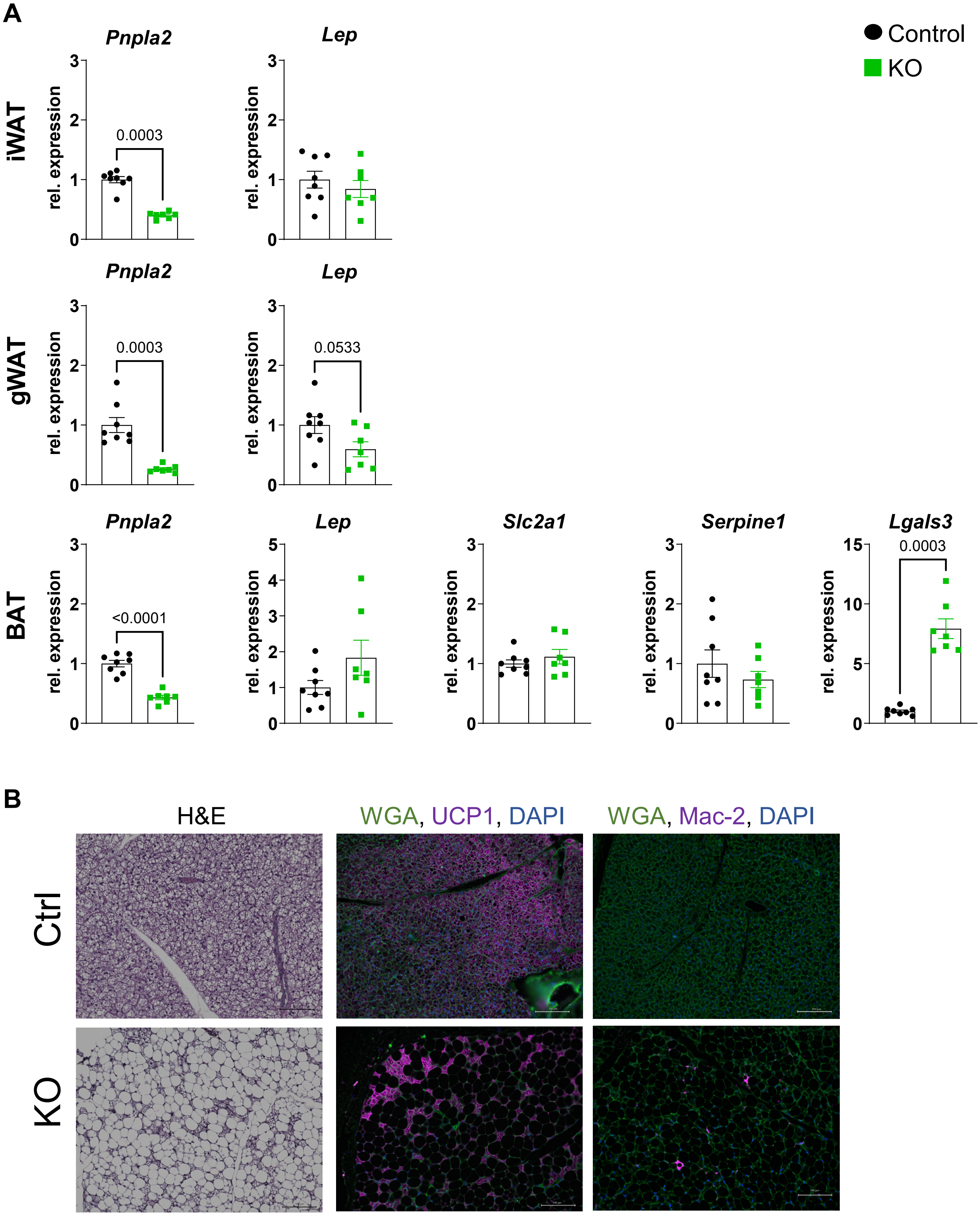

Fig. S10: Adipose tissue biology at baseline. A) Gene expression of ATGL (*Pnpla2*) and leptin (*Lep*) in iWAT and gWAT and *Slc2a1, Serpine1* and *Lgals3* in BAT of KO relative to control at baseline. Data are mean ± SEM, unpaired two-tailed t-test, n= 7-8 B) Histology of BAT: H&E staining, UCP1 and Mac-2 immunofluorescence staining at baseline.

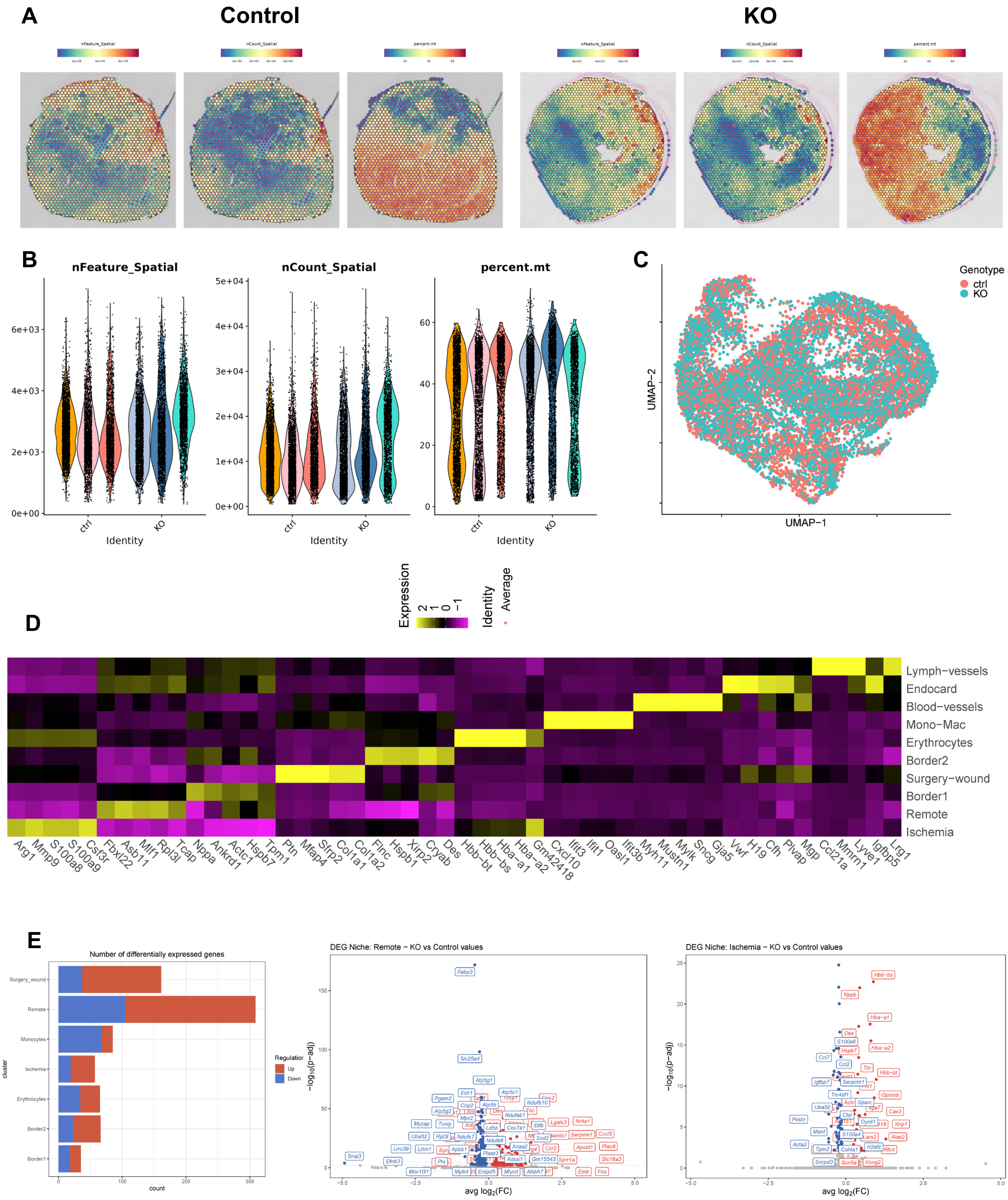

Fig. S11: Quality control of spatial transcriptomic data. A) nFeatures, nCounts and mitochondrial content on exemplary spatial plots of control and KO hearts. B) nFeatures, nCounts and mitochondrial content for all analysed samples (n=3). C) Distribution of genotypes in UMAP plot of clustering. D) Heatmap of top 5 niche markers. E) Number of differentially expressed genes per niche. F) Volcano plots of differentially expressed genes in KO vs. control in niches „Remote“ and „Ischemia“. $p<0.05$, log2FC > 0.25.

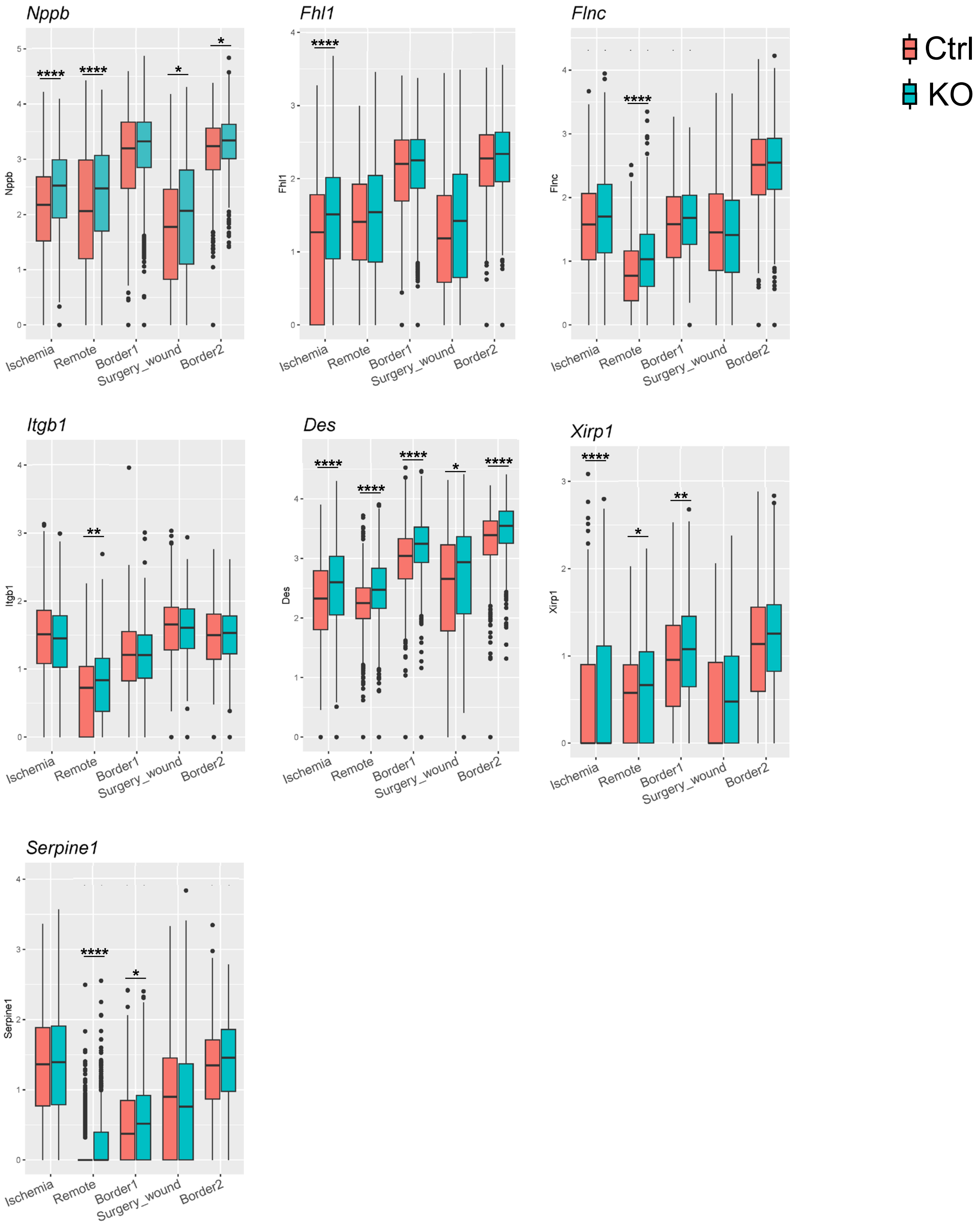

Fig. S12: Gene expression level of mechanical stress associated genes *Nppb, Fhl1, Flnc*, *Des, Itgb1* and *Xirp1* as well as *Serpine1* in different niches. *p<0.05, **p<0.01, ***p<0.001, ****p<0.0001.

A

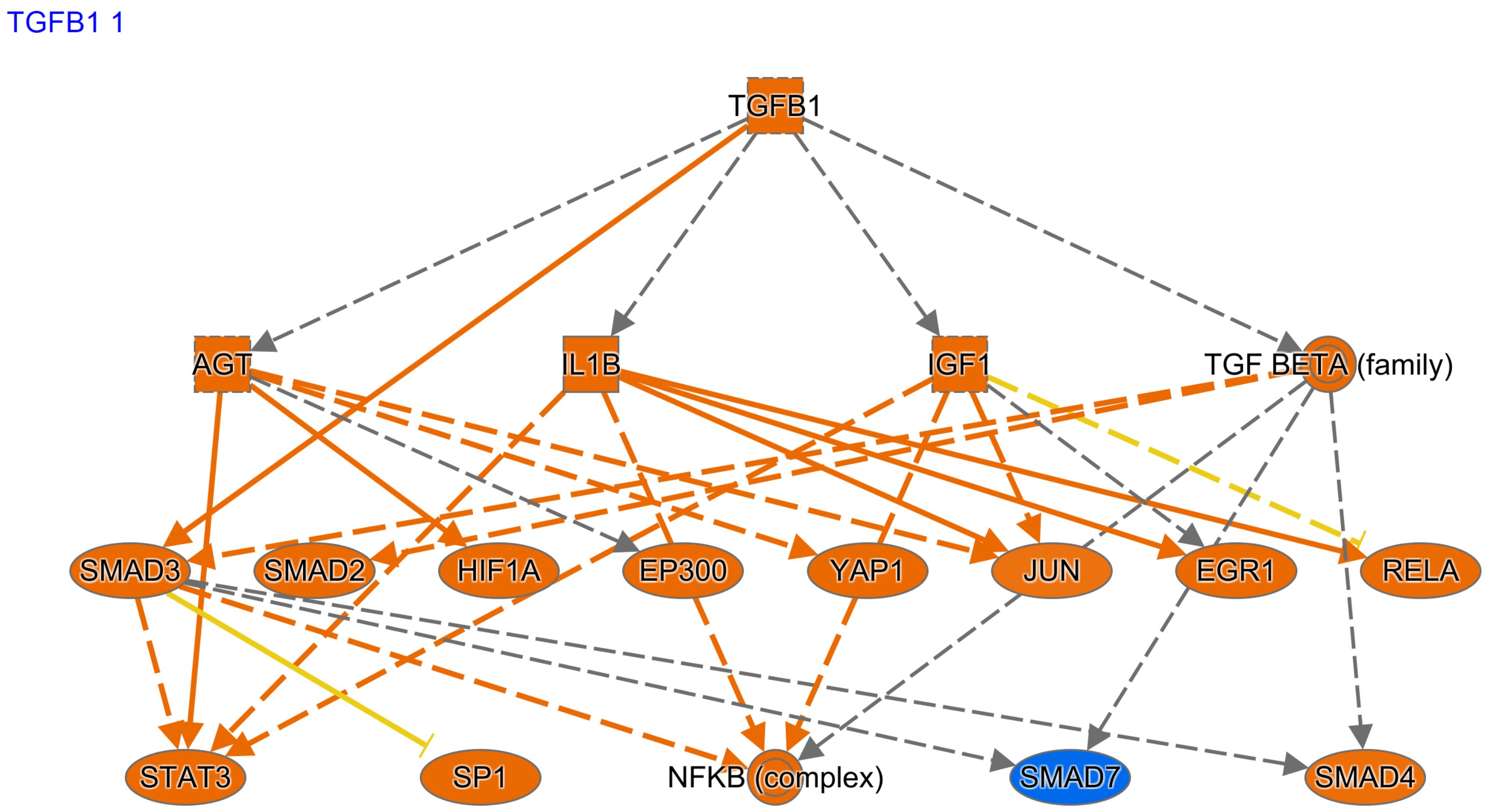

© 2000-2025 QIAGEN. All rights reserved.

B

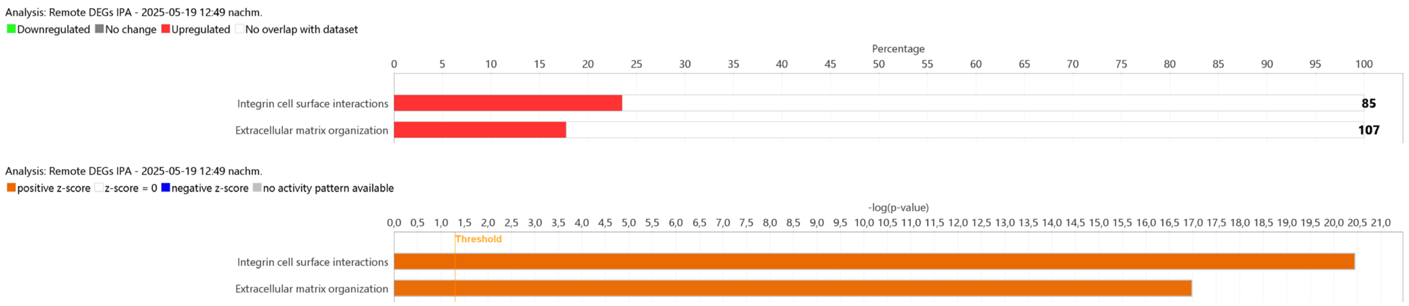

Ingenuity Canonical Pathways	-log(p-value)	Ratio	z-score	Down-regulated	No change	Upregulated	No overlap with dataset	Molecules
Integrin cell surface interactions	2,04E01	2,35E-01	4,472	0/85 (0%)	0/85 (0%)	20/85 (24%)	65/85 (76%)	CD44,COL1A1,COL1A2,COL3A1,COL4A1,COL4A2,COL5A1,COL5A2,COL5A3,COL8A1,FBN1,FN1,HSPG2,ITGA5,ITGA7,ITGB1,ITGB2,SPP1,THBS1,TNC
Extracellular matrix organization	1,7E01	1,78E-01	4,359	0/107 (0%)	0/107 (0%)	19/107 (18%)	88/107 (82%)	BGN,COL1A1,COL1A2,COL3A1,COL4A1,COL4A2,COL5A1,COL5A2,COL5A3,FN1,HSPG2,ITGA5,ITGA7,ITGB1,SERPINE1,SPARC,TGFB2,TNC,VCAN

Fig. S13: Ingenutiy pathway analysis of remote-niche DEGs. A) TGFB1 as main predicted underlying regulator. B) Top two canonical pathways.

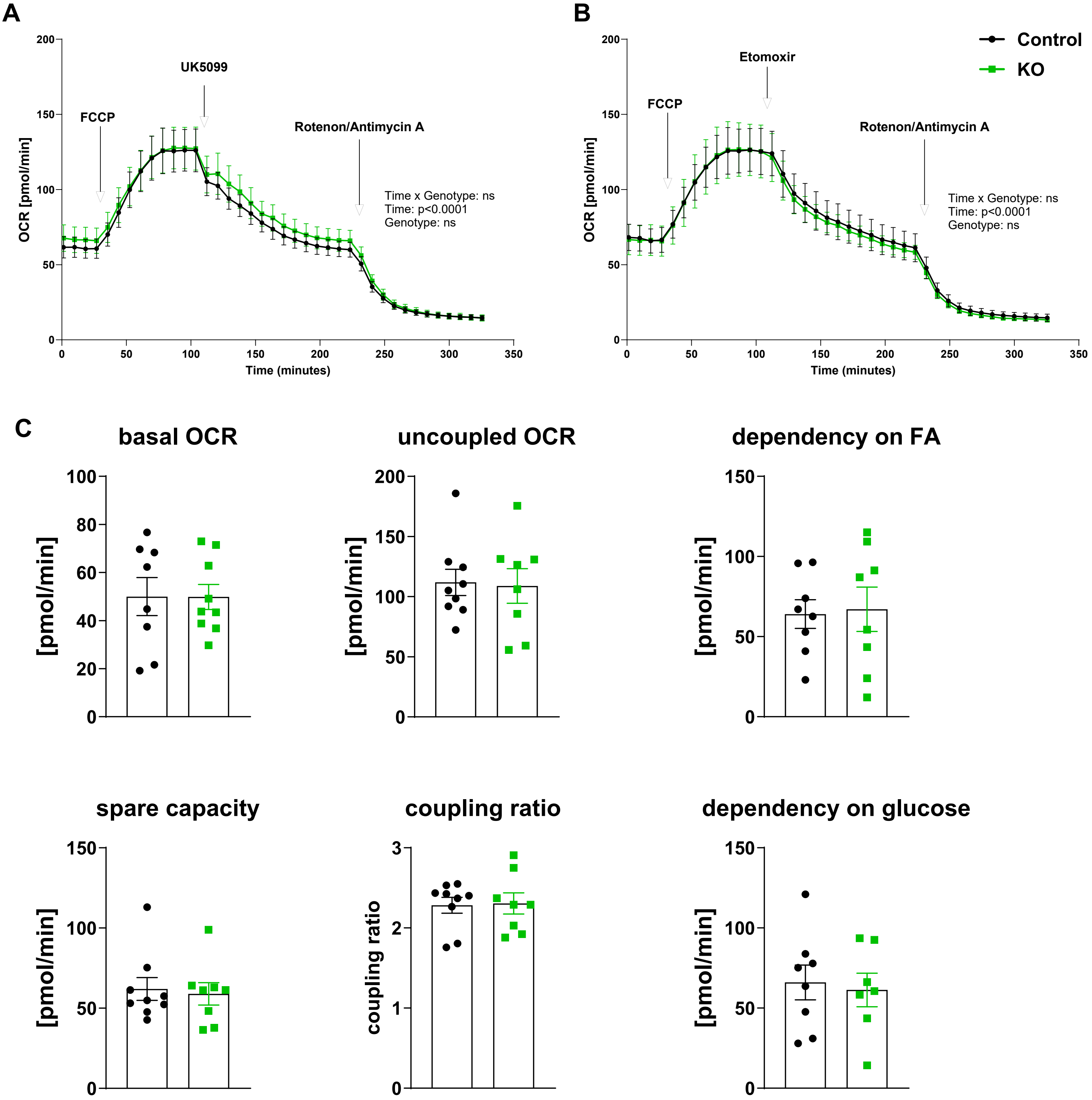

Fig S14: Extracellular flux measurements in cardiac tissue at baseline. n=7-8, data are mean ± SEM, two-way ANOVA (A) and unpaired two-tailed t-test (B).
